# Extended treatment of multimodal cognitive behavioral therapy in children and adolescents with obsessive–compulsive disorder improves symptom reduction: a within-subject design

**DOI:** 10.1186/s13034-022-00537-z

**Published:** 2022-12-09

**Authors:** Julia Adam, Hildegard Goletz, Stefanie Dengs, Nora Klingenberger, Sonja Könnecke, Christina Vonderbank, Christopher Hautmann, Martin Hellmich, Julia Plück, Manfred Döpfner

**Affiliations:** 1 School of Child and Adolescent Cognitive Behavior Therapy (AKiP), Faculty of Medicine and University Hospital Cologne, University of Cologne, Pohligstr. 9, 50969 Cologne, Germany; 2 Institute of Medical Statistics and Computational Biology, Faculty of Medicine and University Hospital Cologne, University of Cologne, Robert-Koch-Str. 10, 50931 Cologne, Germany; 3 Department of Child and Adolescent Psychiatry, Psychosomatics and Psychotherapy, Faculty of Medicine and University Hospital Cologne, University of Cologne, Robert-Koch-Str. 10, 50931 Cologne, Germany

**Keywords:** Obsessive–compulsive disorder, Cognitive behavioral therapy, Exposure with response prevention, Children, Adolescents, Treatment evaluation

## Abstract

**Background:**

Based on the current state of research regarding the treatment in pediatric obsessive–compulsive disorder (OCD), cognitive behavioral therapy (CBT) (in severe cases with additional pharmacotherapy) is considered as the first-line treatment according to internationally recognized guidelines. Research is mostly based on randomized controlled trials (RCTs; efficacy research). Thus, examined treatment conditions, especially the treatment duration, and patients’ characteristics do not necessarily correspond to those found within routine care. Studies showed CBT packages as a whole to be efficacious, but less is known about the effects of individual CBT components. Furthermore, effects on comorbid symptoms or psychosocial impairment have been often neglected and different rater perspectives have been hardly considered in previous research.

**Methods:**

This effectiveness study aimed to examine the effects of multimodal CBT in children, adolescents, and young adults (age 6–20 years) with OCD (*n* = 38) within routine care. Effects on obsessive–compulsive and co-existing symptoms were evaluated in a within-subject design by comparing changes during the assessment phase with 12-week standard treatment and with individually tailored extended treatment. Additionally, within the standard treatment, non-exposure treatment was compared to exposure treatment. Multi-informant assessment was applied, and the analyses included multilevel modeling and t-tests for pre-post comparisons.

**Results:**

During the standard treatment and extended treatment, obsessive–compulsive symptoms, strain, and functional impairment significantly decreased. Moreover, a significant reduction of overall comorbid symptoms emerged, particularly regarding internalizing symptoms, including anxiety and depression. Comparisons of treatment components indicated that adding exposure with response prevention (ERP) has an additional positive effect. Clinical improvement and remission rates increased considerably when more treatment sessions were provided.

**Conclusions:**

These results suggest that improvement after an initial 12-week course of treatment may not allow for the prediction of non-responders/non-remitters and for the termination of treatment. Overall, the findings show that results from randomized controlled trials are transferrable to routine care.

*Trial registration number* This study was registered retrospectively at the German Clinical Trials Register (https://drks.de/search/de/trial/DRKS00030050).

**Supplementary Information:**

The online version contains supplementary material available at 10.1186/s13034-022-00537-z.

## Background

The number of treatment outcome studies for pediatric obsessive–compulsive disorder (OCD) has increased in recent years. On the whole, the study findings demonstrate the efficacy of cognitive behavioral therapy (CBT) and pharmacotherapy in reducing OCD symptoms as well as the superiority of CBT compared to medication alone [1–4]. A combination of pharmacotherapy and CBT has also shown better results than pharmacotherapy as an individual treatment [2, 5, 6]. Based on these studies, CBT (in severe cases with additional pharmacotherapy) is considered as the first-line treatment according to internationally recognized guidelines [7, 8].

Nevertheless, there are still some issues regarding treatment research in pediatric OCD that need to be further investigated:

Most of the reported CBT effects are based on change scores and effect sizes. These do not necessarily describe the clinical relevance of post-treatment OCD symptoms, such as end-state functioning and extent of recovery, which are of particular interest for patients, parents, and clinicians [9]. Some studies investigated remission, reporting rates of 50 to 60% (e.g. [3, 4]). Thus, despite large pre-post effect sizes, almost half of patients still suffer from OCD symptoms in a clinical range at post-treatment. Moreover, barely any studies have examined rates of reliable change as defined by Jacobson & Truax [10].

Furthermore, the majority of studies analyzed CBT packages as a whole, which include several treatment components like psychoeducation, exposure with response prevention (ERP), and parent management training. As such, there is only limited evidence regarding the “active ingredients” of the treatment (e.g. [11]). A small number of studies focusing on individual CBT components showed that both CBT variants (cognitive therapy and ERP) result in significant reductions in OCD severity [12–14]. In contrast to previous meta-analyses, Rosa-Alcázar et al. [15] demonstrated that the most promising treatment packages are those which include ERP, cognitive strategies and relapse prevention.

Meta-analyses by Abramowitz et al. [9]), Sánchez-Meca et al. [2] and Rosa-Alcázar et al. [15] found that CBT also has effects on co-existing symptoms such as anxiety and depression as well as functional impairment. However, most research projects only evaluated the treatment effects on OCD symptoms, while the effects on comorbid symptoms or psychosocial impairment have been neglected. It is especially important to investigate the effects of CBT on psychosocial functioning and other OCD-related problems given that patients with OCD suffer severe functional impairment [16] and show high comorbidity rates, especially with anxiety and depressive symptoms (e.g. [17, 18]).

Moreover, Abramowitz et al. [9] pointed out that most of the OCD-related outcome measures in studies published in recent years were interviewer-based. However, the need for multimodal assessment integrating parents’ and patients’ perspectives is stressed due to low correlations between these raters (e.g. [19, 20]).

The current state of research is mostly based on randomized controlled trials (RCTs; efficacy research), but efficacy research usually includes highly selective study samples. It is therefore questionable whether the samples examined are representative of “real patient populations”, because among other things, patients are usually recruited through advertisements and not spontaneously referred for treatment [21]. Moreover, such trials exclude patients with comorbidities commonly associated with OCD like depressive disorders, or patients with previous treatment attempts [22]. Therefore, the following question arises: To what extent can results from efficacy studies be generalized to routine clinical practice? (e.g. [21, 23, 24]). There are at least some studies examining the effectiveness of manual-based CBT in clinical routine care, which demonstrated treatment effects on pediatric OCD comparable to those from RCTs [25–29].

As a further shortcoming, the treatments evaluated to date have a median duration of approximately 12 weeks and a total duration of around 15.5 h [15], which does not correspond to the average number of intervention hours (27 to 55) implemented in psychotherapy treatment as usual [30]. Therefore, effects of extended treatments are largely unknown, although some studies have reported evidence in this regard. For instance, Sánchez-Meca et al. [2] showed that the magnitude of interventions (total number of treatment hours) was associated with larger effect sizes. Several recent studies demonstrated the effect of extended treatments beyond a treatment length of 7 weeks [31] and 14 weeks [32] and on long-term stability [33]. The present study aimed to systematically examine the effects of (a) a standard 12-week treatment period with the two treatment phases non-exposure and exposure CBT, and (b) an extended treatment option for children, adolescents, and young adults with insufficient symptom improvement. Thus, the CBT treatment was examined in a broad sample including the range of ages (6–20 years) encountered within routine care in children and adolescents. The standardized treatment was tailored individually regarding treatment duration and depending on age and problem constellation, the involvement of the parents and the chosen therapeutic materials could vary. The effects were assessed with (c) multiple-informant outcome measures regarding (d) OCD, comorbid symptoms and functional impairment in (e) patients referred to a university-based outpatient clinic (routine care). Additionally, clinical significance, including remission rates and reliable changes, were investigated.

## Methods

### Inclusion criteria

The study included children, adolescents and young adults (possible age: 4–21 years) with an ICD-10 diagnosis of OCD (F42.x), assessed in a semi-structured clinical interview with the patient and the parents using the *Diagnostic Checklist for OCD* (DCL-ZWA; [34]). Moreover, OCD severity had to be constantly high during the six-week assessment phase (t0-t1; see “Study design and treatment” section), as measured by the *German version of the Children’s Yale-Brown Obsessive–Compulsive Scale* (*CY-BOCS-D*; [35]) and at least in a moderate range (CY-BOCS-D total score ≥ 16; [36]). OCD had to be the primary diagnosis according to clinical judgement, and other symptoms were not allowed to be more prominent, but cases with comorbid disorders were not excluded. Comorbid symptoms were assessed based on standardized questionnaires (see Table 1). OCD-specific medication was allowed if no change in dosage or medication was planned throughout the study period. Further inclusion criteria were IQ ≥ 80 assessed with a standardized intelligence test, outpatient CBT had, according to clinical judgement, to be feasible and indicated, no other psychotherapy was permitted throughout study participation, and patients and parents had to provide written informed consent for study participation.

**Table 1:** Outcomes & multi-informant assessment

Assessment area and assessment points	Patient-rating	Parent-rating	Therapist-rating (administered by the treating therapist)
OCD symptoms & severity			
• Pre-treatment (t0 and t1) and after every sixth weekly treatment session (t2-tx)	OCD-CA	OCD-CA	CY-BOCS-D
• Pre- and post-treatment (t0 and tx)			DCL-ZWA
OCD-related individual problems			
• Pre-treatment (t0 and t1) and every treatment week from t1 onwards	OCD-PL Daily Observation	OCD-PL Daily Observation	
Functional impairment			
• Pre-treatment (t0 and t1) and every treatment week from t1 onwards	OCD-FL	OCD-FL	
Overall comorbid symptoms			
• Pre- and post-treatment (t1 and tx)	YSR	CBCL	
Anxiety			
• Pre- and post-treatment (t1 and tx)	SBB-ANZ	FBB-ANZ	
Depression			
• Pre- and post-treatment (t1 and tx)	SBB-DES	FBB-DES	

*OCD-CA* German OCD Inventory for Children and Adolescents, *CY-BOCS-D* German version of the Children’s Yale-Brown Obsessive–Compulsive Scale, *DCL-ZWA* Diagnostic Checklist for OCD, *OCD-PL* OCD-related problem list, *OCD-FL* OCD-functional impairment list, *YSR* Youth Self Report/ 11-18R, *CBCL* Child Behavior Checklist/ 6-18R, *SBB-ANZ & FBB-ANZ* German Symptom Checklists for Anxiety Disorders and Obsessive–Compulsive Disorders, *SBB-DES & FBB-DES* German Symptom Checklists for Depressive Disorders

### Participant recruitment

Patients were recruited during their initial consultation at the School for Child and Adolescent Cognitive Behavior Therapy at the University Hospital Cologne. All patients had been referred to the outpatient clinic within routine care. If OCD symptoms were prominent, patients and parents were informed about the study and asked to participate. Patients were included between August 2010 and January 2016.

### Study design and treatment

The effectiveness of the treatment (Additional file 1) was tested in a within-subject control group design (Additional file 2) comprising three phases, each with a duration of six weeks (phase 1: assessment; phase 2: standard treatment consisting of phase 2a including non-exposure CBT and phase 2b including exposure CBT) and an extension phase based on the individual needs (phase 3) lasting for 6 to 42 weeks. Thus, the overall treatment period (phase 2 to phase 3) encompassed between 18 and 54 weekly sessions, lasting for 50 min each and up to about 100 min for ERP. Additionally, during the treatment, parent sessions were offered according to the individual problem constellation (every four weeks on average). As soon as the OCD symptoms were in a subclinical range (assessed with the CY-BOCS-D rating scale; [35]; cut-off score ≤ 7; [36, 37]), the treatment was completed with a further six weekly sessions, including multimodal relapse prevention (tx = individual treatment end). Accordingly, treatment end depended on the individual improvement; if OCD symptoms did not sufficiently decrease during CBT, treatment was terminated after 54 weeks (t10 = last possible assessment point).

The manual-based CBT was carried out by educationalists or psychologists who were undergoing three-or-five-year training in child and adolescent psychotherapy. All therapists received two-hour weekly group supervision by the manual’s first author (HG).

### Outcome measures

Table 1 presents an overview of the multi-informant assessment instruments used within the present study. A detailed description is provided in Additional file 3. The primary outcome was OCD severity, derived from the clinician-rated German version of the *Children’s Yale-Brown Obsessive–Compulsive Scale* (*CY-BOCS-D*; [35]). OCD was diagnosed based on the clinician-rated *Diagnostic Checklist for OCD* (*DCL-ZWA*), which is part of the Diagnostic System for the Assessment of Mental Disorders in Children and Adolescents based on the ICD-10 and DSM-IV (DISYPS-II; [34]). Further secondary outcomes were parent- and patient-rated OCD symptoms (*German OCD Inventory for Children and Adolescents* [*OCD-CA*]; [38]), OCD-related individual problems (*OCD-related problem list* [*OCD-PL*]; [38] and *Daily Observation*; [39]), functional impairment (*OCD-functional impairment list,* [*OCD-FL*]; developed for the purpose of this study), overall comorbid symptoms (*Child Behavior Checklist/6-18R* [*CBCL/6-18R*] and *Youth Self Report/11-18R* [*YSR/11-18R*]; [40]), anxiety symptoms (*German Symptom Checklists for Anxiety Disorders and Obsessive–Compulsive Disorders* [*FBB-/SBB-ANZ*]; [34]), and depressive symptoms (*German Symptom Checklists for Depressive Disorders* [*FBB-/SBB-DES*]; [34]).

### Statistical analyses

For the analyses, if less than 10% of the items were missing, only scale values were computed. Intention-to-treat analyses were conducted. First, the within-subject control group [41] design was analyzed using multilevel analysis [42, 43]. Two different analysis models were computed. Time was coded such that the growth rate reflected the estimated weekly change. Model 1 included six time periods, for which growth rates *β* (mean change per week) were calculated: (1) assessment (t0-t1), (2) standard treatment (t1-t3) and (3) extended treatment (t3-t10) divided into phase 3a (t3-t5), phase 3b (t5-t7), phase 3c (t7-t9), and phase 3d (t9-t10, last assessment point). Model 2 comprised seven time periods, as in contrast to model 1, standard treatment (t1-t3) was subdivided into non-exposure CBT (t1-t2) and exposure CBT (t2-t3).

To consider the variability of the individual OCD symptoms and related problems at pre-treatment, the models’ intercept was assumed to be random and slopes were fixed. All cases, including incomplete cases, remained in the analyses [44]. This strategy has been shown to be appropriate if missing data are at random [45]. Data were collected until the individual end of treatment (tx, max. t10); observation was not carried forward until t10 (last possible assessment point) for every case. The criterion for missing data at random is fulfilled because the propensity for data to be missing is related to observed data, the CY-BOCS-D rating scale value [46]. Missing values were not imputed.

The objectives of the analyses (model 1) were to check whether changes during standard treatment (β_standard treatment_) and optional extended treatment (β_extended treatment_) were significant and whether changes during standard treatment (β_standard treatment_) were significantly larger than changes during the assessment phase (β_assessment_). Furthermore, growth rates β_standard treatment_ and growth rates β_extended treatment_ were compared for those patients who received extended treatment.

Moreover, the objective of the analyses with model 2 was to compare differential effects of CBT packages, hypothesizing that changes during exposure CBT in the standard treatment (β_exposure CBT_) would be significantly larger than changes during the preceding non-exposure CBT (β_non-exposure CBT_). T-tests were used for comparisons of assessment phase and standard treatment (β_assessment_ vs. β_standard treatment_) as well as for comparisons of CBT duration and contents (β_standard treatment_ vs. β_extended treatment_; β_non-exposure CBT_ vs. β_exposure CBT_).

Effect sizes (ES) were calculated using the growth rate multiplied by the length of respective time periods (the number of time periods / intervals) and divided by the initial standard deviations (t0).

Second, dependent t-tests for pre-post comparison were calculated if instruments were only used at pre-treatment (t0 or t1) and individual post-treatment (tx, see “Study design and treatment” section). In such cases, ES were computed by calculating the difference between pre- and post-treatment divided by the initial standard deviation (t0 or t1).

Clinical significance was computed according to Jacobson and Truax [10] and Jacobson et al. [47]: (1) To evaluate whether OCD symptoms were in a clinical or subclinical range after 12 standard treatment weeks (t3) and at individual post-treatment (tx), OCD symptoms were classified as clinical or subclinical at these assessment points on the basis of available cut-off values (CY-BOCS-D: total score ≥ 8; [36, 37]). (2) To evaluate whether the extent of change between t0 and t3 as well as between t0 and tx was statistically reliable, the reliable change index (RCI; Jacobson & Truax [10]) was calculated. Subsequently, patients were classified into six groups regarding their change during treatment and status at post-treatment: (1) worsened & clinical range, (2) unchanged & clinical range, (3) worsened & subclinical range, (4) unchanged & subclinical range, (4) improved & clinical range, and (6) improved & subclinical range.

## Results

### Participants

**Fig. 1:**
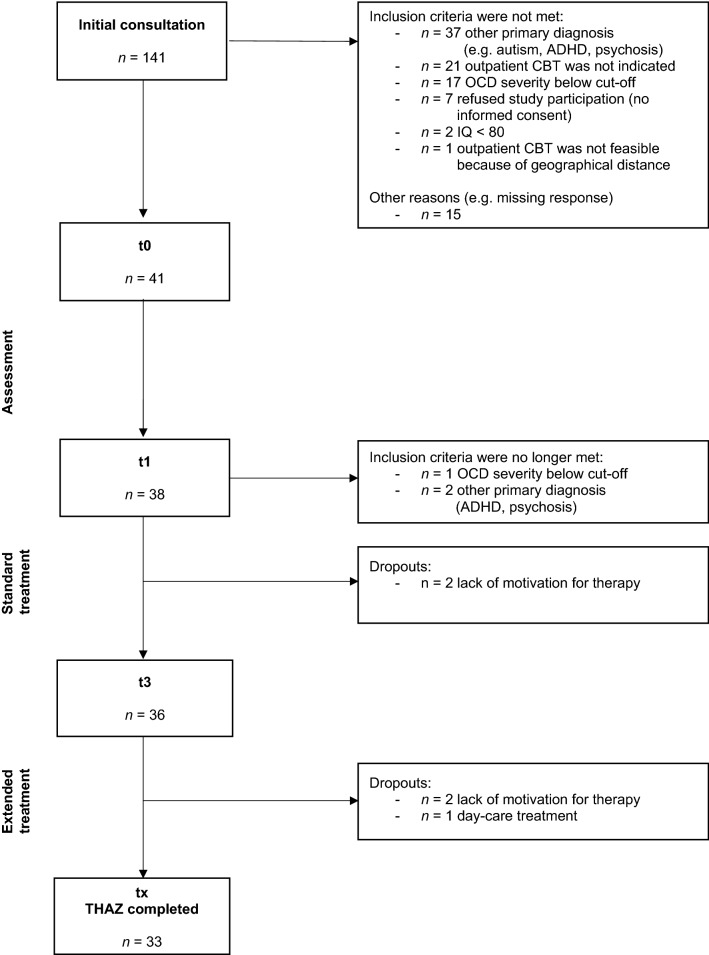
Flow chart

The participant flow of the study is shown in Fig. 1. A total of 38 patients were eligible to participate, 33 of whom finished treatment per protocol.

Table 2 summarizes the demographic and clinical characteristics of the sample. Patients were aged 6 to 20 years (*M* = 13.28, *SD* = 3.56) and 42.1% were boys. On average, OCD symptoms were in a severe range (*M* = 25.05, *SD* = 4.26 [36];). Four patients were receiving OCD-specific medication and 23.7% had comorbid disorders.

**Table 2:** Description of the sample (n = 38)

Age in years: mean (*SD*), range	13.28 (3.56), 6.50–20.17
Male gender: *n* (%)	16 (42.1)
OCD diagnosis: *n* (%)	38 (100)
• Predominantly obsessional thoughts or ruminations (F42.0)	3 (7.9)
• Predominantly compulsive acts, obsessional rituals (F42.1)	8 (21.1)
• Mixed obsessional thoughts and acts (F42.2)	27 (71.1)
OCD severity (CY-BOCS-D rating scale total score): mean (*SD*), range	25.05 (4.26), 17–33
Comorbid diagnoses: *n* (%)	9 (23.7)
• Mild or moderate depressive episode (F32.0, F32.1)	5 (13.2)
• Attention deficit disorder (F98.8)	2 (5.3)
• Combined vocal and multiple motor tic disorder (F95.2)	1 (2.6)
• Separation anxiety disorder of childhood (F93.0)	1 (2.6)
OCD-specific medication: *n* (%)	4 (10.5)

### Treatment effects

The overall treatment duration (phase 2 to phase 3, see “Study design and treatment” section) of patients who finished treatment per protocol ranged from 18 to 54 weekly sessions (*M* = 41.09, *SD* = 14.24). Thus, all patients needed extended treatment (see Additional file 4). Table 3 shows results for the slopes (growth rates) and effect sizes of the assessment, standard treatment, and extended treatment phases as well as results of the comparisons of these phases with one another regarding OCD symptoms, strain, and impairment.

**Table 3:** Results of multilevel analyses: Assessment (t0-t1) vs. standard treatment (t1-t3) vs. extended treatment (t3-t10)

		Change during assessment		Change during standard treatment		Change during extended treatment							
		Phase 1: t0-t1		Phase 2 and 3: t1-t3		Phase 3a: t3-t5		Phase 3b: t5-t7		Phase 3c: t7-t9		Phase 3d: t9-t10	
Outcome	*n*	*β (CI 95%)*	*ES*	*β (CI 95%)*	*ES*	*β (CI 95%)*	*ES*	*β (CI 95%)*	*ES*	*β (CI 95%)*	*ES*	*β (CI 95%)*	*ES*
CY-BOCS-D rating scale													
	**38**	**− 0.34**^**a**^** (− 0.69 to 0.01)**	**− 0.48**	− 0.54***^b,c^** (− 0.72 to − 0.36)**	**− 1.53**	− 0.42***^a,c^** (− 0.61 to − 0.24)**	**− 1.19**	**− 0.05**^**b,d**^** (− 0.25 to 0.16)**	**− 0.14**	**− 0.08**^**b,d**^** (− 0.33 to 0.16)**	**− 0.23**	**− 0.06**^**a,c**^** (− 0.62 to 0.49)**	**− 0.09**
Obsession severity	38	− 0.18^a^ (− 0.40 to 0.04)	− 0.25	− 0.23***^a,c^ (− 0.35 to − 0.12)	− 0.66	− 0.17**^a,c^ (− 0.28 to − 0.05)	− 0.47	− 0.07^a,d^ (− 0.19 to 0.06)	− 0.18	0.01^b,d^ (− 0.14 to 0.16)	0.03	− 0.13^a,c^ (− 0.47 to 0.21)	− 0.18
Compulsion severity	38	− 0.18*^a^ (− 0.36 to − 0.00)	− 0.49	− 0.29***^b,c^ (− 0.38 to − 0.20)	− 1.56	− 0.22***^a,c^ (− 0.32 to − 0.13)	− 1.21	0.01^b,d^ (− 0.09 to 0.11)	0.05	− 0.06^b,d^ (− 0.18 to 0.07)	− 0.31	− 0.05^a,c^ (− 0.32 to 0.23)	− 0.12
OCD-CA													
Total OCD symptoms	[31]	[− 0.32^a^] [(− 1.20 to 0.56)]	[− 0.11]	[− 0.53*^a,c^] [(− 0.97 to − 0.10)]	[− 0.38]	[− 0.65**^a,c^] [(− 1.09 to − 0.21)]	[− 0.46]	[− 0.42^a,c^] [(− 0.91 to 0.06)]	[− 0.30]	[− 0.03^a,c^] [(− 0.59 to 0.53)]	[− 0.02]	[0.70^a,c^] [(− 0.61 to 2.01)]	[0.25]
	{37}	{− 0.72^a^} {(− 1.48 to 0.04)}	{− 0.25}	{− 0.56**^a,c^} {(− 0.96 to − 0.16)}	{− 0.40}	{− 0.73***^a,c^} {(− 1.13 to − 0.32)}	{− 0.51}	{− 0.03^b,d^} {(− 0.48 to 0.43)}	{− 0.02}	{− 0.09^b,c^} {(− 0.62 to 0.45)}	{− 0.06}	{0.25^a,c^} {(− 0.98 to 1.49)}	{0.09}
OCD-related problem list													
Frequency	[31]	[− 0.04*^a^] [(− 0.08 to − 0.00)]	[− 0.23]	[− 0.05***^a,c^] [(− 0.06 to − 0.04)]	[− 0.57]	[− 0.04***^a,c^] [(− 0.05 to − 0.03)]	[− 0.47]	[− 0.02***^b,d^] [(− 0.03 to − 0.01)]	[− 0.25]	[− 0.01^b,d^] [(− 0.03 to 0.00)]	[− 0.17]	[0.06**^b,d^] [(0.02 to 0.10)]	[0.36]
	{32}	{− 0.01^a^} {(− 0.05 to 0.04)}	{− 0.04}	{− 0.06***^b,c^} {(− 0.08 to − 0.04)}	{− 0.90}	{− 0.04***^b,d^} {(− 0.06 to − 0.02)}	{− 0.62}	{0.00^a,d^} {(− 0.02 to 0.02)}	{− 0.05}	{− 0.01^a,d^} {(− 0.03 to 0.01)}	{− 0.16}	{− 0.02^a,c^} {(− 0.08 to 0.05)}	{− 0.14}
Strain	[31]	[− 0.12**^a^] [(− 0.21 to − 0.03)]	[− 0.39]	[− 0.07***^b,c^] [(− 0.10 to − 0.04)]	[− 0.46]	[− 0.11***^a,d^] [(− 0.14 to − 0.09)]	[− 0.73]	[− 0.04**^b,c^] [(− 0.07 to − 0.01)]	[− 0.28]	[− 0.01^b,d^] [(− 0.05 to 0.02)]	[− 0.09]	[0.01^b,d^] [(− 0.05 to 0.16)]	[0.18]
	{32}	{− 0.13*^a^} {(− 0.25 to − 0.01)}	{− 0.49}	{− 0.11***^a,c^} {(− 0.15 to − 0.06)}	{− 0.81}	{− 0.10***^a,c^} {(− 0.14 to − 0.06)}	{− 0.77}	{0.00^b,d^} {(− 0.04 to 0.05)}	{0.04}	{− 0.03^b,d^} {(− 0.09 to 0.03)}	{− 0.25}	{0.09^b,d^} {(− 0.07 to 0.25)}	{0.34}
Psychosocial impairment	[30]	[− 0.06***^a^] [(− 0.10 to − 0.03)]	[− 0.36]	[− 0.03***^b,c^] [(− 0.04 to − 0.02)]	[− 0.36]	[− 0.02***^b,c^] [(− 0.03 to − 0.01)]	[− 0.25]	[− 0.01^b,d^] [(− 0.02 to 0.01)]	[− 0.06]	[0.00^b,d^] [(− 0.01 to 0.01)]	[0.00]	[0.00^b,c^] [(− 0.03 to 0.04)]	[0.03]
	{36}	{− 0.03^a^} {(− 0.07 to 0.01)}	{− 0.26}	{− 0.05***^b,c^} {(− 0.07 to − 0.04)}	{− 0.82}	{− 0.02*^b,d^} {(− 0.03 to − 0.00)}	{− 0.28}	{0.00^b,d^} {(− 0.02 to 0.02)}	{0.05}	{0.00^b,d^} {(− 0.02 to 0.03)}	{0.04}	{− 0.04^a,c^} {(− 0.10 to 0.02)}	{− 0.29}
OCD-functional impairment list													
Total psychosocial impairment	[30]	[− 1.35***^a^] [(− 1.64 to − 1.05)]	[− 0.57]	[− 0.36***^b,c^] [(− 0.46 to − 0.27)]	[− 0.31]	[− 0.26***^b,d^] [(− 0.35 to − 0.18)]	[− 0.22]	[− 0.10*^b,d^] [(− 0.19 to − 0.01)]	[− 0.09]	[− 0.17**^b,d^] [(− 0.28 to − 0.05)]	[− 0.14]	[0.27^b,d^] [(− 0.08 to 0.61]	[0.11]
	{35}	{− 1.52***^a^} {(− 2.01 to − 1.03)}	{− 0.80}	{− 0.56***^b,c^} {(− 0.75 to − 0.37)}	{− 0.59}	{− 0.19*^b,d^} {(− 0.38 to 0.00)}	{− 0.20}	{− 0.03^b,d^} {(− 0.25 to 0.18)}	{− 0.04}	{− 0.43**^b,c^} {(− 0.70 to − 0.16)}	{− 0.45}	{0.13^b,c^} {(− 0.60 to 0.86)}	{0.07}

*n* sample size, *β* slope, *CI* confidence interval, *ES* effect size; bold values show the results of the primary outcome; clinical rating, [self-report], {parent report};*p≤.05, **p≤.01, ***p≤.001; ^a,b,c,d^slopes with superscripts (a) do not differ significantly from assessment phase, slopes with superscript (b) differ significantly at a level of ≤.05 from assessment phase; slopes with superscripts (c) do not differ significantly from standard treatment phase, slopes with superscript (d) differ significantly at a level of ≤.05 from standard treatment phase

**Fig. 2:**
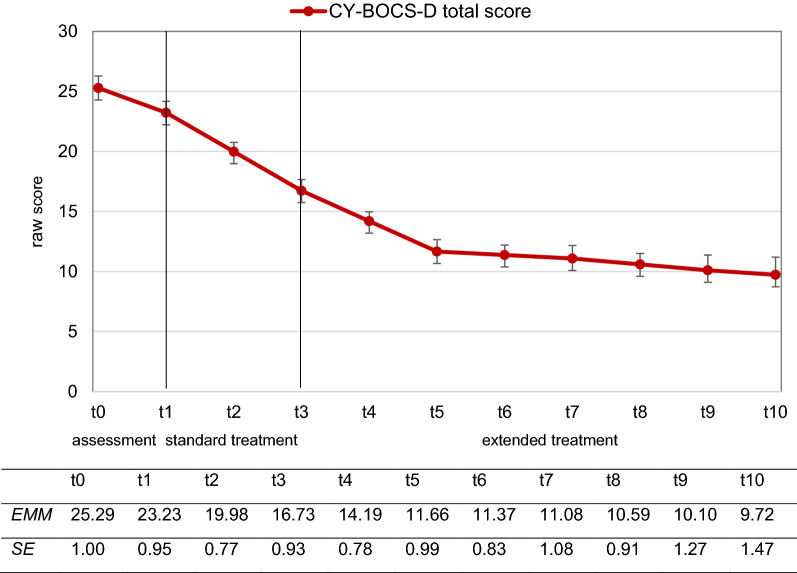
Estimated course of OCD severity (CY-BOCS-D). *EMM* estimated marginal mean, *SE* standard error

On the primary outcome (CY-BOCS-D rating scale), the clinician-rated total OCD severity (see also Fig. 2) did not significantly decrease during the assessment (*A*) phase (*β* = − 0.34, *p* = 0.056, *ES* = − 0.48). During standard treatment (*ST*), there was a significant mean decrease per week (*β* = − 0.54, *p* ≤ 0.001) and the effect (*ES* = − 1.53, *Δ ES*_*A-ST*_ = 1.05) was considerably larger compared to the assessment phase. Considering the entire extended treatment (*ET*; t3-t10), the effect was also large (ES = − 1.65, *Δ ES*_*ST-ET*_ = 0.12) between treatment weeks 12 and 54 (phase 3a—phase 3d). A more detailed analysis, however, revealed that only during treatment weeks 12 to 24 (phase 3a) did clinician-rated OCD severity significantly decrease (*β* = − 0.42, *p* ≤ 0.001), with a large effect size (ES = − 1.19). Growth rates (mean changes per week) and effects during further extended treatment (phase 3b – phase 3d) were only (very) small. Comparable results emerged for the CY-BOCS-D subscales assessing clinician-rated obsession and compulsion severity.

Complementary analyses on secondary outcomes revealed the following findings (Table 3, Additional file 5):

During the assessment phase, growth rates (β_assessment_) of patient- and parent-rated OCD-specific outcomes mainly did not differ significantly from zero, indicating that patient- and parent-rated OCD symptoms (OCD-CA), OCD frequency (OCD-PL), extent of negative emotions, and OCD duration (Daily Observation) were relatively stable during the assessment phase without any treatment. However, psychosocial impairment resulting from OCD symptoms (OCD-PL, OCD-FL, Daily Observation) decreased significantly during the assessment phase (with the exception of parent-rated psychosocial impairment assessed with the OCD-PL). With regard to strain resulting from OCD symptoms, a significant decrease during the assessment phase was found in parent-ratings, while the results of patient-ratings were inconsistent (no significant change, significant decrease) across measures (OCD-PL, Daily Observation).

During standard treatment, patient- and parent-rated total OCD symptoms (OCD-CA) showed a significant reduction. Regarding extended treatment, a significant reduction of patient- and parent-rated OCD symptoms was only found during treatment weeks 12 to 24 (phase 3a), comparable to clinical ratings. Effects during standard treatment and extended treatment were smaller than clinician-rated effects on OCD symptoms and mainly in the small to moderate range. On all other secondary outcomes, significant decreases during standard treatment were found. Moreover, for almost all secondary outcomes, significant decreases were also apparent during the first 12 extended treatment weeks (phase 3a). However, OCD-related problems only partially significantly decreased during further extended treatment phases, and no further significant decrease was found during treatment weeks 48 to 54 (phase 3d). While effect sizes during standard treatment were predominantly in the moderate to large range, effect sizes during separate extended treatment phases were mainly small to moderate.

Despite (considerably) larger effect sizes on almost all secondary outcomes during standard treatment compared to the assessment phase (*Δ* ES_A-ST_), significant differences in growth rates (mean change per week) were only found for some outcomes (parent-ratings of OCD symptom frequency and psychosocial impairment [OCD-PL], patient-rated extent of negative emotions on weekdays, patient-rated strain on weekdays and weekends, patient- and parent-rated OCD duration on weekdays, and patient-rated OCD duration on weekends [Daily Observation]). Conversely, on several outcomes regarding strain and psychosocial impairment, the mean change per week (growth rate) was even significantly larger during assessment than during the standard treatment phase (patient-rated strain [OCD-PL], patient- and parent-rated psychosocial impairment [OCD-FL, OCD-PL], parent-rated extent of negative emotions on weekends, and parent-rated strain on weekdays and weekends [Daily Observation]).

The comparison of the course of patient-rated and parent-rated OCD symptoms and related problems during standard treatment and extended treatment (phase 2 vs. phase 3a-3d) revealed the following: Considering the entire extended treatment phase, additional absolute effects were comparable to the absolute effects of the standard treatment. Considering extended treatment phases separately, the only phase that partially kept up with the mean change per week and absolute effects of the standard treatment was the extended treatment phase 3a (treatment weeks 12–24). On the whole, improvement (mean change per week) during extended treatment phases 3b-3d (treatment weeks 24–54) was significantly smaller than improvement during the standard treatment phase.

Overall, both for the primary outcome and for most complementary analyses of OCD-related variables, moderate to strong effects were found during the standard treatment phase, while small to moderate effects emerged during the extended treatment phases. Most of the improvement in OCD symptoms and related problems occurred during standard treatment and the first 12 extended treatment weeks. During the subsequent extended treatment weeks (phase 3b – phase 3d), the mean change per week and therefore change and absolute effects were mainly much smaller.

Further complementary analyses of pre- and post-ratings (Table 4) of comorbid symptoms showed a significant reduction across the entire treatment phase (with individually tailored treatment duration) on the following: clinician-rated OCD-associated personality traits; patient- and parent-rated overall comorbid problems (CBCL, YSR total problems), and particularly internalizing problems (CBCL, YSR); patient- and parent-rated anxiety and depressive symptoms according to ICD-10/DSM-IV; and parent-rated competences (FBB-/SBB-ANZ, FBB-/SBB-DES), with effect sizes in the small to large range.

**Table 4:** Results of complementary pre-post comparisons on comorbid symptoms

Outcome		*n*	Pre *M (SD)*	Post *M (SD)*	*t*	*ES*
Personality traits DCL-ZWA	Personality traits	20	0.90 (0.59)	0.32 (0.54)	3.59**	− 0.98
Overall comorbid symptoms [YSR] {CBCL}	Internalizing problems	[22]	[9.91 (9.33)]	[7.23 (8.82)]	[2.38*]	[− 0.29]
		{25}	{12.60 (7.36)}	{7.96 (6.94)}	{3.85***}	{− 0.63}
	Externalizing problems	[22]	[6.41 (4.94)]	[5.95 (7.44)]	[0.46]	[− 0.09]
		{25}	{8.20 (5.45)}	{6.04 (6.62)}	{1.84}	{− 0.40}
	Total problems	[22]	[30.68 (20.95)]	[22.86 (22.43)]	[2.91**]	[− 0.37]
		{25}	{35.96 (19.48)}	{24.72 (20.33)}	{3.58**}	{− 0.58}
Anxiety symptom severity & competences [SBB-ANZ] {FBB-ANZ}	Total anxiety	[24]	[0.54 (0.42)]	[0.29 (0.29)]	[4.03***]	[− 0.60]
		{26}	{0.57 (0.39)}	{0.33 (0.33)}	{4.13***}	{− 0.62}
	Competences	[24]	[1.60 (0.62)]	[1.60 (0.81)]	[0.04]	[0.00]
		{25}	{1.56 (0.51)}	{1.82 (0.63)}	{− 2.84**}	{0.51}
Depressive symptom severity & competences [SBB-DES] {FBB-DES}	Total depressive symptoms	[25]	[0.39 (0.42)]	[0.21 (0.34)]	[3.47**]	[− 0.43]
		{27}	{0.39 (0.25)}	{0.23 (0.22)}	{3.81***}	{− 0.64}
	Competences	[25]	[1.96 (0.77)]	[1.98 (0.86)]	[− 0.12]	[0.03]
		{27}	{1.75 (0.63)}	{1.96 (0.68)}	{− 3.19**}	{0.33}

*n* sample size, *M*  mean, *SD* standard deviation, *t* t-test for dependent samples, *ES* effect size, clinical rating, [self-report], {parent report}, ***p ≤ 0.001 **p ≤ 0.01 *p ≤ 0.05

Results of comparisons between CBT components (β_non-exposure CBT_ vs. β_exposure CBT_) are presented in Additional files 6, 7 and 8.

On the primary outcome (CY-BOCS-D rating scale), there was a significant decrease in clinician-rated total OCD severity during both phases (non-exposure CBT [NE]: *β* = − 0.46, *p* = 0.016; exposure CBT [E]: *β* = − 0.62, *p* ≤ 0.001), while no significant difference regarding growth rates was found. However, considering the total effects, compared to the moderate effect during non-exposure CBT (*ES* = − 0.65), a large effect during exposure CBT (*ES* = − 0.87, *Δ ES*_*NE-E*_ = 0.22) was found. Regarding CY-BOCS-D subscales, there were no differences in growth rate and effect sizes on the clinician-rated obsession severity subscale, but differences did emerge on the clinician-rated compulsion severity subscale, suggesting that exposure CBT has particular effects on compulsions.

When significant differences in secondary outcomes were found between phases, these were in favor of the exposure CBT (with the only exception being patient-rated duration of OCD symptoms on weekdays with the Daily Observation). The clearest result emerged regarding the extent of negative emotions (Daily Observation; Additional file 7): In particular, the decrease in the extent of negative emotions was significantly larger in patient- and parent-ratings during exposure CBT than during non-exposure CBT.

### Clinical significance and reliable change

The mean percentage reduction in the CYBOCS-D rating scale total score (primary outcome) from baseline (t0; *M* = 25.05, *SD* = 4.26) to post-treatment (tx, individual treatment end; *M* = 7.82, *SD* = 6.39) was 68.8%. After 12 treatment weeks (t3, *M* = 16.53, *SD* = 6.66), percentage reduction in the CYBOCS-D rating scale total score was 34%.

**Fig. 3:**
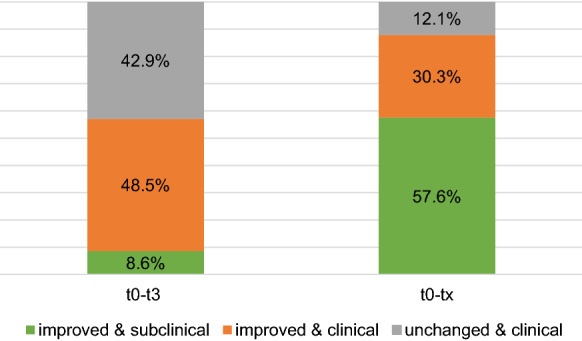
Clinical significance of change in clinician-rated OCD severity (CY-BOCS-D)

As large effect sizes do not necessarily indicate subclinical posttest symptomatology, clinical significance was investigated in order to assess patients’ end-state functioning and recovery. The results are presented in Fig. 3.

None of the children and adolescents showed a clinically significant deterioration regarding clinician-rated OCD severity (CY-BOCS-D rating scale) after the standard treatment and at the individual end of treatment. While after the first 12 treatment weeks, 42.9% of the sample were still in a clinical range and unchanged, after extended treatment, this proportion lay at only 12.1%.

On the clinician-rated CY-BOCS-D rating scale, 57.1% of the sample were significantly improved after standard treatment, and 8.6% of the sample were also in a subclinical range. After extended treatment, the improvement rate (87.9%) and especially normalization (57.6% subclinical) was considerably higher.

## Discussion

The present effectiveness study aimed to investigate the course of OCD symptoms as well as psychosocial impairment and comorbid symptoms during a cognitive behavioral intervention for children and adolescents diagnosed with OCD within a regular outpatient setting. A special focus was on the effects of differential CBT packages (non-exposure CBT vs. exposure CBT) and individually tailored treatment duration (standard treatment vs. extended treatment). Moreover, clinical significance was investigated.

Overall, the results revealed a significant improvement during the standard treatment phase (first 12 weekly sessions) and the first extended treatment phase (treatment weeks 12–24) on the primary outcome (clinician-rated CY-BOCS-D) and on almost all OCD-specific and OCD-related outcomes, including functional impairment and strain. Effect sizes during the standard treatment phase and the entire extended treatment phase were mainly moderate to large, while effects during separate extended treatment phases were small to moderate. Benchmarking (Table 5) shows that changes in clinician-rated total OCD symptoms during standard treatment (*ES* = 1.53) and the entire extended treatment (*ES* = 1.65) are widely comparable to within-group effect sizes reported in other effectiveness studies [26–28] and to effect sizes that considered effects of control groups reported in efficacy studies [2, 3]. In contrast to other effectiveness studies [25–28] as well as efficacy studies [2, 3], the outcome measures in the current study were not only clinician-administered. Rather, we computed effect sizes separately for the clinician-, patient-, and parent-ratings. With regard to OCD symptoms, changes based on clinician-ratings (*ES* = − 1.53, − 1.65; overall: *ES* = − 3.18) were considerably higher than those based on patient-ratings (*ES* = − 0.38, − 0.53; overall: *ES* = − 0.91) and parent-ratings (*ES* = − 0.40, − 0.50; overall: *ES* = − 0.90). Rosa-Alcázar et al. [15] found comparable differences in their meta-analysis when computing effect sizes separately for rater perspectives (clinician-report: *ES* = 2.02; patient-report: *ES* = 0.82). There are several potential explanations for these findings. Patients might show dissimulation tendencies or may not report their symptoms accurately due to embarrassment about their OCD, in particular at pre-treatment [20]. Parents may underestimate their children’s symptoms, because some symptoms (in particular obsessions) are more difficult for them to notice [48]. Furthermore, as the treating therapist in the present study was also the clinician rater, a rater bias may have occurred due, for instance, to justifying one’s own efforts but also due to higher sensitivity of therapist-rating. Moreover, differences between outcome measures have to be taken into account. While the clinician-rated CY-BOCS-D focuses on global OCD severity (including impairment, resistance and control), the patient- and parent-rated OCD-CA focuses on OCD symptoms in different domains without considering impairment, resistance and control [49].

**Table 5:** Benchmarking: Comparison of study results with findings of different efficacy studies (meta-analyses) and effectiveness studies

	Efficacy studies (Meta-analyses)		Effectiveness studies				
Study	Sánchez-Meca et al. [2]	McGuire et al. [3]	Valderhaug et al. [25]	Nakatani et al. [26]	Farrell et al. [27]	Torp et al. [28]	Current study
Completers *%*	*M* = 91.4 (treatment group)	Range of means: 73 – 100 (treatment group)	86.0	Not available	94.3	89.6	86.8
Mean reduction in CY-BOCS total score *%*	Not investigated	Not investigated	After 12 sessions: 60.6	After 5–28 sessions: 51.8	After 8–14 sessions: 61	After 14 sessions: 52.9 (SD = 30.9)	After 12 sessions: 34.0 after 18–54 sessions: 68.8
Effect size on total OCD symptoms	Global: after *M* = 11.8 weeks: 1.7^1^	Clinician-rated: after 9–14 sessions: 1.2^1^	Clinician-rated: after 12 sessions: 3.5^2^	Clinician-rated: after 5–28 sessions: 2.3^2^	Clinician-rated: after 8–14 sessions: 2.1^2^	Clinician-rated: after 14 sessions: 1.6^2^	Clinician-rated: after 12 sessions: 1.5^2^ after further 42 sessions: 1.7^2^ child-rated: after 12 sessions: 0.4^2^ after further 42 sessions: 0.5^2^ parent-rated: after 12 sessions: 0.4^2^ after further 42 sessions: 0.5^2^
Remission criteria & rate *%*	Not investigated	Criteria: no consistent remission criteria among RCTs (e.g. CY-BOCS ≤ 10 or CY-BOCS ≤ 14)	Criteria: CY-BOCS ≤ 9	Criteria: CY-BOCS ≤ 11	Criteria: CY-BOCS ≤ 10	Criteria: CY-BOCS ≤ 10	Criteria: CY-BOCS ≤ 7 (CY-BOCS ≤ 10)
		Remission rate: after 9–14 sessions: 57	Remission rate: after 12 sessions: 50	Remission rate: after 5–28 sessions: 60	Remission rate: after 8–14 sessions: 63	Remission rate: after 14 sessions: 49.4	Remission rate: after 12 sessions: 8.6 (16.7) after 18–54 sessions: 57.6 (57.6)
Reliable change % based on CY-BOCS	Not investigated	Not investigated	Not investigated	Not investigated	After 8–14 sessions: 86	After 14 sessions: 70.1	After 12 sessions: 57.1 after 18–54 sessions: 87.9
Effects on comorbid symptoms	After *M* = 11.8 weeks: Anxiety: Global: *ES* = 0.6^1^ (*n* = 6 studies) Depression: Global*: ES* = 0.4^1^ (*n* = 6 studies)	Not investigated	Not investigated	Not investigated	After 8–14 sessions: Overall comorbid symptoms: Clinician-rated: 45% reduction in secondary diagnoses Anxiety: Child-rated: *ES* = 0.2^2^, 0.4^2^ Depression: Child-rated: *ES* = 0.3^2^	Not investigated	After 18–54 sessions: Overall comorbid symptoms: child-rated: *ES* = 0.4^2^ parent-rated: *ES* = 0.6^2^ Anxiety: child-rated: *ES* = 0.6^2^ parent-rated: *ES* = 0.6^2^ Depression: clinician-rated: *ES* = 0.5^2^ child-rated: *ES* = 0.4^2^ parent-rated: *ES* = 0.6^2^
Effects on psychosocial impairment	Global: after *M* = 11.8 weeks: *ES* = 0.8^1^ (*n* = 4 studies)	Not investigated	Child-rated: after 12 sessions: 49.6% mean reduction Parent-rated: after 12 sessions: 60.8% mean reduction	Not investigated	Child-rated: after 8–14 sessions: *ES* = 0.5^2^ Parent-rated: after 8–14 sessions: *ES* = 0.5^2^	Not investigated	Child-rated: after 12 sessions: *ES* = 0.3^2^ after further 42 sessions: *ES* = 0.3^2^ Parent-rated: after 12 sessions: *ES* = 0.6^2^ after further 42 sessions: *ES* = 0.6^2^

^1^Standardized mean difference between the change scores of the treatment and the control groups, ^2^Standardized mean difference pre-post-treatment

While changes in clinician-rated OCD symptoms during standard treatment are comparable to benchmarks (see Table 5), the mean reduction in the CY-BOCS total score (34%) is considerably lower than the values reported in the other effectiveness studies (e.g. 60.6%; [25]). However, the mean reduction in the CY-BOCS-D total score reached at individual end of treatment (68.8%) is even higher than the values reported in other effectiveness studies (Table 5).

It is generally problematic to compare remission rates across different studies. Despite efforts to standardize the criteria for remission (e.g. [50, 51]), the criteria employed vary across studies. The CY-BOCS cut-off criterion of ≤ 7 used in the current study is stricter than that used in other studies. Thus, we additionally computed the remission rate based on a CY-BOCS cut-off criterion of ≤ 10 for comparison. To summarize, even with this less strict cut-off, the remission rate after standard treatment was considerably lower than those derived from studies within benchmarking, but the remission rates at the individual end of treatment were comparable (see Table 5). Reliable change after individual extended treatment was in line with the results reported by Farrell et al. [27] and Torp et al. [28].

As mentioned above, the mean reduction in the CY-BOCS-D total score and the clinician-rated remission rate after the first 12 treatment weeks were lower than the results of other internationally published studies. This may be attributable to therapist, sample or treatment characteristics. Overall, when comparing the present study with other efficacy and effectiveness studies, some discrepancies are apparent (see Additional file 9). In the present study, exclusion criteria were kept to a minimum. Thus, in contrast to Torp et al. [28], patients with previous CBT attempts were also included, and unlike Valderhaugh et al. [25], no specific psychiatric disorder was excluded. The main differences pertain to the therapist’s experience, which was lower in the present study than in the cited effectiveness studies (with the exception of Farrell et al. [27], in which the level of therapists’ experience was roughly comparable). Furthermore, pre-treatment mean OCD symptoms in the current study were severe (*M* = 25.05), while the assessed OCD severity in the other effectiveness studies (with the exception of Torp et al. [28]; *M* = 24.6) was somewhat lower and in a moderate range (CY-BOCS total score < 24; cut-off criterion according to Bossert-Zaudig & Niedermeier [36]; AACAP [7]). A further key difference lies in the notably longer overall treatment duration (18–54 sessions) in the current study. For example, knowing that a maximum of 54 sessions was possible may have led the therapist to choose smaller steps within graduated ERP, which may have resulted in a slower improvement.

Concerning changes during the treatment of overall comorbid symptoms, significant small to moderate effects were found for total problems and internalizing problems, including anxiety and depressive symptoms (-0.29 ≤ *ES* ≤ -0.64). These findings are in line with Sánchez-Meca et al. [2] and Rosa-Alcázar et al. [15], but the effects are higher than those reported by Farrell et al. [27], and in contrast to Abramowitz et al. [9], whose combined effect size for anxiety and depressive symptoms was not statistically significant. As expected, no significant effects were found on externalizing problems. Effects on psychosocial impairment are broadly in accordance with previous findings (Table 5).

During the assessment phase, a stable course or increase of OCD symptoms and functional impairment was expected, and this expectation applied to most outcomes. However, the clinician-rated compulsion severity (CY-BOCS-D) decreased significantly during the assessment phase, and this was also the case for the majority of patient- and parent-rated strain and psychosocial impairment outcomes. When comparing growth rates between assessment and standard treatment phase, significant differences in favor of the standard treatment phase for clinician-rated total OCD severity (CY-BOCS-D) and some other outcomes (e.g. OCD duration on weekdays) were found, as well as greater absolute effects. This result did not emerge, for instance, for the patient- and parent-rated total psychosocial impairment with the OCD-PL (on which significant differences in favor of the assessment phase were found) and total OCD symptoms (no significant differences between phases were found). These findings lead to the impression that unspecific effects were active during the assessment phase. The significant decrease especially in functional impairment and strain during the assessment phase might be explained, for instance, by the feeling of being understood by the therapist or by positive expectations of treatment (e.g. [52]). Nevertheless, it is unlikely that the described unspecific effects occurring during the assessment phase would continue for a further 18 to 54 weeks and that only conducting assessment sessions would therefore be as effective as treatment sessions.

The comparison of CBT packages revealed some significant differences in favor of exposure CBT. Accordingly, there is at least some support for an additional effect of ERP. The clearest findings emerged from the analyses regarding the extent of negative emotions. This was to be expected given that ERP aims especially at habituation, and thus a correction of physiological components of the negative emotion (extinction processes) caused by the OCD-triggering situations or thoughts, but also aims at fear tolerance [53]. As only six treatment weeks of each CBT package were compared within this study, it can only be assumed that the tendency found might be even clearer when comparing longer treatment durations of each package.

The main conclusion derived from the comparison of CBT durations was that absolute effects of the standard treatment are comparable with the additional absolute effects of the extended treatment (treatment weeks 12–54; phase 3a – phase 3d). However, most change / improvement in OCD symptoms and related problems occurred during standard treatment and the first 12 extended treatment weeks. During the following extended treatment weeks, the mean change per week and therefore change and absolute effects were mainly much smaller.

Overall, these findings regarding treatment duration support the relevance of individually tailored and extended treatment. In line with the findings of Torp and Skarphedinsson [31] and Skarphedinsson et al. [32], the present results suggest that improvement after the initial course of CBT may not allow for treatment termination. Rather, our findings suggest that substantial improvement mainly occurs during the first 24 weekly CBT sessions. Accordingly, improvement and potential further extension of treatment should particularly be found after about six months of treatment. If a patient has not substantially improved by treatment week 24, for instance, treatment motivation or strategies should be questioned. Corresponding to our findings, in particular after 48 weekly sessions, there is a tendency that may suggest that no further (substantial) improvement can be expected. In the present study, we did not investigate potential factors that may explain and predict individually required treatment duration as well as treatment success. Further research to investigate this issue would be interesting. Skarphedinsson et al. [32] identified barriers to treatment success during the initial course of CBT, for example, “patient took long time to engage and start exposure exercises due to high levels of anxiety or low motivation” or “family factors, such as high initial accommodation”. Melin et al. [33] found a higher level of symptoms at baseline in non-responders than in responders to be the only significant group difference in an initial course of CBT.

A main limitation is that the clinician rater was the treating therapist. The lack of blinded and independent clinician-ratings should not only be noted when comparing rater perspectives, but above all, when comparing effects to other effectiveness studies, which used predominantly blinded or at least independent evaluators [25, 27, 28]. However, patients and parents were blinded to the specific hypotheses regarding treatment contents and duration. Moreover, Lewin et al. [54] showed that therapists might even represent a reasonable alternative to blind and independent evaluators to rate pediatric OCD improvement.

Although the exclusion criteria were kept to a minimum, the rate of comorbid disorders in the present sample (23.7%) does not correspond to the high comorbidity rates, ranging from 62 to 97%, found in children and adolescents with OCD [17, 55]. This low comorbidity rate may be due on the one hand to the inclusion criterion that OCD had to be the primary diagnosis, or on the other hand to the lack of systematic assessment of comorbidities. While individual comorbid symptoms were assessed by parent- and patient-ratings, clinical diagnoses of comorbid disorders were not systematically confirmed by structured interviews. Considering the parent- and patient-ratings revealed the following: While patients > 11 years reported low comorbidity within the YSR assessment (12.9%, valid percentage: 15.4%), the comorbidity rate reported by parents was much higher. Within the CBCL, 47.4% (valid percentage: 58.1%) of the patients showed comorbid symptoms in a clinical range (at least one subscale or the total scale was in a clinical range; the subscale thought problems was excluded from this analysis because it comprises items regarding OCD symptoms). This parent-reported comorbidity rate is widely comparable to those reported by other effectiveness studies (Additional file 9).

To conclude, the comorbidity rate in the study sample may presumably be higher than reported. Nevertheless, the representativeness regarding comorbidities remains questionable.

Another principal limitation of this study is that it does not constitute an RCT. As such, it cannot be ruled out that external factors may have been responsible for the treatment outcome. However, given that the explicit aim of this study was to evaluate the effectiveness of manualized CBT, the fact that it was not an RCT, and the effects were not investigated under laboratory conditions, constitutes a strength at the same time. In contrast to RCTs, the emphasis was on external validity and not on internal validity [23]. Moreover, the chosen within-subject control group design maintained at least a certain level of internal validity, and patients served as their own control group, leading to a reduced error variance [56]. The within-subject analyses are also conservative, since they assume that a trend observed during the waiting phase would also continue during the consecutive treatment phases.

While the present study aimed to evaluate manual-based treatment under routine care conditions, it is rather questionable whether the supervision conducted within this study (and other effectiveness studies) can be achieved under non-research or routine conditions [28]. Thus, it remains unclear whether the treatment conditions of effectiveness studies are entirely comparable to non-research and “real-life” conditions.

Another limitation is that the research team included authors of the evaluated treatment program. Therefore, the possibility of researcher allegiance cannot be ruled out, and a replication of the findings by other researchers is therefore warranted.

Finally, the large number of outcome variables in the exploratory analyses increases the likelihood of incidental findings. However, besides treatment effects on OCD, effects on impairment and comorbidities were hypothesized, and a respective number of measures was required to test these hypotheses across different rater perspectives.

## Conclusion

Overall and despite some limitations, the present study contributes further to “bridging the gap between laboratory and clinic” [21]. The results support the effectiveness of manualized exposure-based CBT in children, adolescents, and young adults with OCD in terms of reducing OCD symptoms, psychosocial impairment, overall comorbid symptoms, and in particular internalizing problems, including anxiety and depressive symptoms. Moreover, the effectiveness was confirmed by multiple informants. To conclude, results from RCTs seem to be transferrable to “real-world” clinical settings and generalizable to routine clinical practice. Importantly, the present findings provide evidence in favor of individually tailored treatment durations.

## Supplementary Information

**Additional file 1. **Details of the treatment. Details of the CBT treatment are described.

**Additional file 2. **Within-subject design clinical trial. The research design is presented in a figure.

**Additional file 3.** Outcome measures. The outcome measures used within the study are described.

**Additional file 4.** Individual end of treatment. The individual end of treatment as well as dropouts are presented in a figure.

**Additional file 5.** Results of multilevel analyses: Assessment (t0-t1) vs. treatment (t1-t3) vs. extended treatment (t3-t10). Changes during assessment phase and the treatment phases regarding the daily observation are shown in a table.

**Additional file 6.** Results of multilevel analyses: Assessment (t0-t1) vs. non-exposure CBT (t1-t2) vs. exposure CBT (t2-t3) vs. extended treatment (t3-t10). Changes during assessment phase and the treatment phases as well as effects regarding the clinician-rated OCD severity, patient- and parent-rated OCD symptoms and OCD-related problems are presented in a table.

**Additional file 7.** Results of multilevel analyses: Assessment (t0-t1) vs. non-exposure CBT (t1-t2) vs. exposure CBT (t2-t3) vs. extended treatment (t3-t10). Changes during assessment phase and the treatment phases as well as effects regarding daily observation are shown in a table.

**Additional file 8. **Results of multilevel analyses: Assessment (t0-t1) vs. non-exposure CBT (t1-t2) vs. exposure CBT (t2-t3) vs. extended treatment (t3-t10). Changes during assessment phase and the treatment phases as well as effects regarding OCD functional impairment are presented in a table.

**Additional file 9.** Benchmarking: Study characteristics. Characteristics of efficacy studies (Meta-analyses) and effectiveness studies are summarized in a table for benchmarking.

## Data Availability

The datasets used and analyzed during the current study are available from the corresponding author on reasonable request.

## Abbreviations

OCD: Obsessive–compulsive disorder
CBT: Cognitive behavioral therapy
RCT: Randomized controlled trials
ERP: Exposure with response prevention
POTS: Pediatric OCD Treatment Study
AACAP: American Academy of Child and Adolescent Psychiatry
NICE: National Institute for Health and Care Excellence
ICD-10: Tenth edition of the International Statistical Classification of Diseases and Related Health Problems
DCL-ZWA: Diagnostic Checklist for OCD
CY-BOCS-D: German version of the Children’s Yale-Brown Obsessive–Compulsive Scale
DSM-IV: Fourth edition of the Diagnostic and Statistical Manual of Mental Disorders
DISYPS-II: Diagnostic System for the Assessment of Mental Disorders in Children and Adolescents based on the ICD-10 and DSM-IV
OCD-CA: German OCD Inventory for Children and Adolescents
OCD-PL: OCD-related problem list
OCD-FL: OCD-functional impairment list
CBCL: Child Behavior Checklist
YSR: Youth Self Report
FBB-/SBB-ANZ: Parents- and patient-rated German Symptom Checklists for Anxiety Disorders and Obsessive–Compulsive Disorders
FBB-/SBB-DES: Parents- and patient-rated German Symptom Checklists for Depressive Disorders
ES: Effect size
RCI: Reliable change index
A: Assessment phase
ST: Standard treatment
ET: Entire extended treatment
NE: Non-exposure CBT
E: Exposure CBT

## References

[1] Watson HJ, Rees CS (2008). Meta-analysis of randomized, controlled treatment trials for pediatric obsessive-compulsive disorder. J Child Psychol Psychology.

[2] Sánchez-Meca J, Rosa-Alcázar AI, Iniesta-Sepúlveda M, Rosa-Alcázar Á (2014). Differential efficacy of cognitive behavioral therapy and pharmacological treatments for pediatric obsessive-compulsive disorder: a meta-analysis. J Anxiety Disord.

[3] McGuire JF, Piacentini J, Lewin AB, Brennan EA, Murphy TK, Storch EA (2015). A meta-analysis of cognitive behavior therapy and medication for child obsessive-compulsive disorder: moderators of treatment efficacy, response, and remission. Depress Anxiety.

[4] Öst LG, Riise EN, Wergeland GJ, Hansen B, Kvale G (2016). Cognitive behavioral and pharmacological treatments of OCD in children: a systematic review and meta-analysis. J Anxiety Disord.

[5] Pediatric OCD Treatment Study (POTS) Team (2004). Cognitive-behavior therapy, sertraline, and their combination for children and adolescents with obsessive-compulsive disorder: the Pediatric OCD Treatment Study (POTS) randomized controlled trial. JAMA.

[6] Franklin ME, Sapyta J, Freeman JB, Khanna M, Compton S, Almirall D (2011). Cognitive behavior therapy augmentation of pharmacotherapy in pediatric obsessive-compulsive disorder: the Pediatric OCD Treatment Study II (POTS II) randomized controlled trial. JAMA.

[7] American Academy of Child and Adolescent Psychiatry (AACAP) Committee on Quality Issues (2012). Practice parameter for the assessment and treatment of children and adolescents with obsessive-compulsive disorders. J Am Acad of Child Adolesc Psychiatry..

[8] National Institute for Health and Care Excellence. Obsessive-compulsive disorder and body dysmorphic disorder: treatment. NICE guidelines [CG31]. 2005. https://www.nice.org.uk/guidance/CG31. Assessed 28 Jul 2022. 31869034

[9] Abramowitz JS, Whiteside SP, Deacon BJ (2005). The effectiveness of treatment for pediatric obsessive-compulsive disorder: a meta-analysis. Behav Ther.

[10] Jacobson NS, Truax P (1991). Clinical significance: a statistical approach to defining meaningful change in psychotherapy research. JCCP.

[11] Freeman J, Garcia A, Frank H, Benito K, Conelea C, Walther M, Edmunds J (2014). Evidence based update for psychosocial treatments for pediatric obsessive-compulsive disorder. J Clin Child Adolesc Psychol.

[12] Simons M, Schneider S, Herpertz-Dahlmann B (2005). Metacognitive therapy for pediatric obsessive-compulsive disorder. Psychother Psychosom.

[13] De Haan E, Hoogduin KAL, Buitelaar JK, Keijsers GPJ (1998). Behavior therapy versus clomipramine for the treatment of obsessive-compulsive disorder in children and adolescents. J Am Acad Child Adolesc Psychiatry.

[14] Bolton D, Perrin S (2008). Evaluation of exposure with reponse-prevention for obsessive compulsive disorder in childhood and adolescence. J Behav Ther Exp Psychiatry.

[15] Rosa-Alcázar AI, Sánchez-Meca J, Rosa-Alcázar Á, Iniesta-Sepálveda M, Olivares-Rodríguez J, Parada-Navas JI (2015). Psychological treatment of obsessive-compulsive disorder in children and adolescents: a meta-analysis. Span J Psychol.

[16] Piacentini P, Bergman RL, Keller M, McCracken J (2003). Functional impairment in children and adolescents with obsessive-compulsive disorder. J Child Adolesc Psychopharmacol.

[17] Geller D, Biederman J, Jones J, Park K, Schwartz S, Shapiro S, Coffey B (1998). Is juvenile obsessive-compulsive disorder a developmental subtype of the disorder? A review of the pediatric literature. J Am Acad Child Adolesc Psychiatry.

[18] Jans Th, Wewetzer Ch, Klampfl K, Schulz E, Herpertz-Dahlmann B, Remschmidt H, Warnke A (2007). Phänomenologie und Komorbidität der Zwangsstörung bei Kindern und Jugendlichen. Z Kinder Jugendpsychiatr Psychother.

[19] De Los RA, Augenstein TM, Wang M, Thomas SA, Drabick DAG, Burgers DE, Rabinowitz J (2015). The validity of the multi-informant approach to assessing child and adolescent mental health. Psychol Bull.

[20] Canavera KE, Wilkins KC, Pincus DB, Ehrenreich-May JT (2009). Parent-child agreement in the assessment of obsessive-compulsive disorder. J Clin Child Adolesc Psychol.

[21] Weisz JR (2000). Agenda for child and adolescent psychotherapy research. Arch Gen Psychiatry.

[22] Barrett PM, Farrell L, Pina AA, Peris TS, Piacentini J (2008). Evidence-based psychosocial treatments for child and adolescent obsessive-compulsive disorder. J Clin Child Adolesc Psychol.

[23] Hunsley J, Lee CM (2007). Research-informed benchmarks for psychological treatments: efficacy studies, effectiveness studies, and beyond. Prof Psychol Res Pr.

[24] Lee CM, Horvath C, Hunsley J (2013). Does it work in the real world? The effectiveness of treatments for psychological problems in children and adolescents. Prof Psychol Res Pr.

[25] Valderhaug R, Larsson B, Götestam KG, Piacentini J (2007). An open clinical trial of cognitive-behaviour therapy in children and adolescents with obsessive-compulsive disorder administered in regular outpatient clinics. Behav Res Ther.

[26] Nakatani E, Mataix-Cols D, Micali N, Turner C, Heyman I (2009). Outcomes of cognitive behaviour therapy for obsessive compulsive disorder in a clinical setting: a 10-year experience from a specialist OCD service for children and adolescents. Child Adolesc Ment Health.

[27] Farrell LJ, Schlup B, Boschen MJ (2010). Cognitive-behavioral treatment of childhood obsessive-compulsive disorder in community-based clinical practice: clinical significance and benchmarking against efficacy. Behav Res Ther.

[28] Torp NC, Dahl K, Skarphedinsson G, Thomsen PH, Valderhaug R, Weidle B, Melin KH, Hybel KA, Nissen JB, Lenhard F, Wentzel-Larsen T, Franklin ME, Ivarsson T (2015). Effectiveness of cognitive behavior treatment for pediatric obsessive-compulsive disorder: acute outcomes from the Nordic Long-term OCD Treatment Study (NordLOTS). Behav Res Ther.

[29] Beig I, Döpfner M, Goletz H, Plück J, Dachs L, Kinnen C, Walter D (2017). Alltagswirksamkeit kognitiver Verhaltenstherapie bei Kindern und Jugendlichen mit Zwangsstörungen in einer Ausbildungsambulanz. Z Kinder Jugendpsychiatr Psychother.

[30] Kazdin AE, Bass D, Ayers WA, Rodgers A (1990). Empirical and clinical focus of child and adolescent psychotherapy research. J Consult Clin Psychol.

[31] Torp NC, Skarphedinsson G (2017). Early responders and remitters to exposure-based CBT for pediatric OCD. J Obsessive Compuls Relat Disord.

[32] Skarphedinsson G, Weidle B, Thomsen PH, Dahl K, Torp NC, Nissen JB, Melin KH, Hybel K, Valderhaug R, Wentzel-Larsen T, Compton SN, Ivarsson T (2015). Continued cognitive-behavior therapy versus sertraline for children and adolescents with obsessive-compulsive disorder that were non-responders to cognitive-behavior therapy: a randomized controlled trial. Eur Child Adolesc Psychiatry.

[33] Melin K, Skarphedinsson G, Thomson PH, Weidle B, Torp NC, Valderhaug R, Hojgaard DRMA, Hybel KA, Becker Nissen J, Jensen S, Dahl K, Skärsäter I, Storm Haugland B, Ivarsson T (2020). Treatment gains are sustainable in pediatric obsessive-compulsive disorder: three-year follow-up from the NordLOTS. J Am Acad Child Adolesc Psychiatry.

[34] Döpfner M, Görtz-Dorten A, Lehmkuhl G, Breuer D, Goletz H (2008). Diagnostik-System für psychische Störungen nach ICD-10 und DSM-IV für Kinder und Jugendliche-II (DISYPS-II).

[35] Goletz H, Döpfner M (2018). Die klinische Beurteilung von Zwangssymptomen bei Kindern und Jugendlichen. Eine Studie mit der Children’s Yale-Brown Obsessive-Compulsive Scale (CY-BOCS-D). Z Kinder Jugendpsychiatr Psychother..

[36] Bossert-Zaudig S, Niedermeier N, Zaudig M, Hauke W, Hegerl U (2002). Therapiebegleitende Diagnostik und Messinstrumente bei Zwangsstörungen. Die Zwangsstörung. Diagnostik und Therapie.

[37] Abramowitz JS (2006). Obsessive-compulsive disorder Advances in psychotherapy—evidence-based practice.

[38] Goletz H, Adam J, Döpfner M (2020). DZ-KJ. Diagnostikum für Zwangsstörungen im Kindes- und Jugendalter.

[39] Goletz H, Döpfner M, Roessner V (2018). Zwangsstörungen. Leitfaden Kinder- und Jugendpsychotherapie.

[40] Döpfner M, Plueck J, Kinnen C, Arbeitsgruppe Deutsche Child Behavior Checklist. CBCL Handbuch-Schulalter. Manual zum Elternfragebogen über das Verhalten von Kindern und Jugendlichen, (CBCL/ 6-18R), zum Lehrerfragebogen über das Verhalten von Kindern und Jugendlichen (TRF/6-18R) und zum Fragebogen für Jugendliche (YSR/11-18R). Goettingen: Hogrefe; 2014.

[41] Cotton JW (1998). Analyzing within-subjects experiments.

[42] Raudenbush SW, Bryk AS (2002). Hierarchical linear models: applications and data analysis methods.

[43] Singer JD, Willett JB (2003). Applied longitudinal data analysis: modeling change and event occurrence.

[44] Maas CJM, Snijders TAB (2003). The multilevel approach to repeated measures for complete and incomplete data. Qual Quant.

[45] Rubin DB (1976). Inference and missing data. Biometrika.

[46] Enders CK (2010). Applied missing data analysis.

[47] Jacobson NS, Roberts LJ, Berns SB, McGlinchey JB (1999). Methods for defining and determining the clinical significance of treatment effects: description, application and alternatives. J Consult Clin Psychol.

[48] Rapoport JL, Inoff-Germain G, Weissman MM, Greenwald S, Narrow WE, Jensen PS, Lahey BB, Canino G (2000). Childhood obsessive–compulsive disorder in the NIMH MECA study: parent versus child identification of cases. J Anxiety Disord.

[49] Adam J, Goletz H, Mattausch SK, Plück J, Döpfner M (2019). Psychometric evaluation of a parent-rating and self-rating inventory for pediatric obsessive-compulsive disorder: German OCD Inventory for Children and Adolescents (OCD-CA). Child Adolesc Psychiatry Ment Health.

[50] Storch EA, Lewin AB, De Nadai AS, Murphy TK (2010). Defining treatment response and remission in obsessive-compulsive disorder: a signal detection analysis of the children’s yale-brown obsessive compulsive scale. J Am Acad Child Adolesc Psychiatry.

[51] Skarphedinsson G, De Nadai AS, Storch EA, Lewin AB, Ivarsson T (2017). Defining cognitive-behavior therapy response and remission in pediatric OCD: a signal detection analysis of the Children’s Yale Brown Obsessive Compulsive Scale. Eur Child Adolesc Psychiatry.

[52] Grawe K, Grawe-Gerber M (1999). Ressourcenaktivierung. Psychotherapeut.

[53] Craske MG, Kircanski K, Zelikowsky M, Mystkowski J, Chowdhury N, Baker A (2008). Optimizing inhibitory learning during exposure therapy. Behav Res Ther.

[54] Lewin AB, Peris TS, De Nadai AS, McCracken JT, Piacentini J (2012). Agreement between therapists, parents, patients, and independent evaluators on clinical improvement in pediatric obsessive compulsive disorder. J Consult Clin Psychol.

[55] Wewetzer Ch, Klampfl K, Wewetzer CH (2004). Phänomenologie der juvenilen Zwangsstörung. Zwänge bei Kindern und Jugendlichen.

[56] Gliner JA, Morgan GA, Harmon RJ (2002). Single-factor repeated-measures designs: Analysis and interpretation. J Am Acad Child Adolesc Psychiatry.

