# Supplementary material for: Extended treatment of multimodal cognitive behavioral therapy in children and adolescents with obsessive–compulsive disorder improves symptom reduction: a within-subject design
Source: Child Adolesc Psychiatry Ment Health. 2022 Dec 9;16:99. doi: 10.1186/s13034-022-00537-z (PMC9737735; doi:10.1186/s13034-022-00537-z)
Supplement: Supplementary file 1 — Additional file 1. Details of the treatment. Details of the CBT treatment are described. [file 13034_2022_537_MOESM1_ESM.pdf]

Additional file 1

Details of the treatment

The treatment was based on the German treatment program for children and adolescents with OCD (Therapieprogramm für Kinder und Jugendliche mit Angst- und Zwangsstörungen – Zwänge [THAZ-Zwänge]; Goletz & Döpfner, in prep.).

Contents of non-exposure CBT (phase 2a, t1-t2) were the development of a therapeutic relationship and activation of resources, psychoeducation and enhancement of motivation for therapy, treatment of problem-maintaining familial, school, and other conditions, as well as cognitive interventions regarding dysfunctional cognitions. During the further treatment phases (phase 2b and phase 3, t2-tx), exposure with response prevention (ERP) for the treatment of compulsive acts and obsessive thoughts were added. Furthermore, patients were instructed with weekly homework regarding ERP assignments. The final six weekly sessions also comprised multimodal relapse prevention, including emotion-focused interventions and social skills training. Therapeutic materials within the THAZ treatment program could be tailored individually to the patient according to the mentioned and planned interventions.

Reference

Goletz, H. & Döpfner, M. (in prep.). *Zwangsstörungen: Ein Therapieprogramm für Kinder und Jugendliche mit Angst- und Zwangsstörungen (THAZ) – Band 3*. Göttingen: Hogrefe.
