# Supplementary figures and images for: Extended treatment of multimodal cognitive behavioral therapy in children and adolescents with obsessive–compulsive disorder improves symptom reduction: a within-subject design
Source: Child Adolesc Psychiatry Ment Health. 2022 Dec 9;16:99. doi: 10.1186/s13034-022-00537-z (PMC9737735; doi:10.1186/s13034-022-00537-z)

## Additional file 2

*Within-subject design clinical trial*

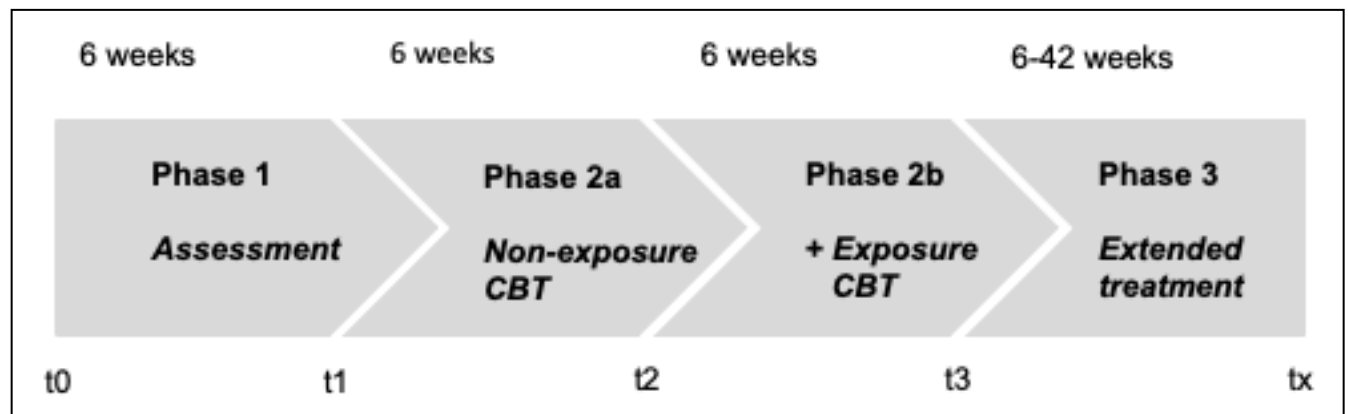

Supplement: Supplementary file 2 — Additional file 2. Within-subject design clinical trial. The research design is presented in a figure. [file 13034_2022_537_MOESM2_ESM.pdf]
