# Supplementary material for: Extended treatment of multimodal cognitive behavioral therapy in children and adolescents with obsessive–compulsive disorder improves symptom reduction: a within-subject design
Source: Child Adolesc Psychiatry Ment Health. 2022 Dec 9;16:99. doi: 10.1186/s13034-022-00537-z (PMC9737735; doi:10.1186/s13034-022-00537-z)
Supplement: Supplementary file 3 — Additional file 3. Outcome measures. The outcome measures used within the study are described. [file 13034_2022_537_MOESM3_ESM.pdf]

Additional file 3
Outcome measures

German version of the Children's Yale-Brown Obsessive-Compulsive Scale (CY-BOCS-D; Goletz & Döpfner, 2018). The CY-BOCS-D is based on the English original version of the CY-BOCS (Goodman et al., 1986). OCD severity is rated based on a parent interview (patients < 11 years) or patient interview (patients ≥ 11 years). The CY-BOCS-D includes a checklist and a rating scale. Within this study, the rating scale (semi-structured interview) was used to measure obsession severity, compulsion severity, and the total OCD severity. The total OCD severity scale was derived by summing up the responses to items 1-10, and obsession and compulsion severity were derived by summing up the responses to items 1-5 and 6-10, respectively (items 1b and 6b were excluded). Items are rated on a 5-point Likert scale (0-4), with higher scores indicating greater symptom severity. The CY-BOCS-D has shown acceptable and good internal consistency, respectively. Sufficient support for the validity of the CY-BOCS-D has also been found (Goletz & Döpfner, 2018).
Diagnostic Checklist for OCD (DCL-ZWA; Döpfner et al., 2008). The checklist includes OCD diagnostic criteria according to ICD-10 and DSM-IV. OCD was diagnosed with this checklist. Furthermore, OCD-associated personality traits (eight items) were assessed on a 5-point scale ("0 = none" to "4 = extreme"). As the psychometric properties of this DCL-ZWA scale have not been evaluated so far, internal consistency was examined in the study sample, showing an acceptable result (α = .72).For scale formation, the item values are added up and divided by the number of items.
German OCD Inventory for Children and Adolescents (OCD-CA; Goletz, Adam & Döpfner, 2020).
The OCD-CA is a modified version of the Padua Inventory – Washington State University Revision (PI-WSUR; Burns et al., 1996 / PI-WSUR (German translation); Department for Neuropsychology of the University Hospital Bonn, 2002). It comprises two multidimensional questionnaires, a parent form (6 to 18 years) and a self-report form (11 to 18 years). Both questionnaires include the same 36 items for assessing various obsessions and compulsions on a 5-point scale from 0 (not at all) to 4 (very much). The OCD-CA total scale was used for the analyses. For scale formation, ratings of the items are added up. The OCD-CA was found to be a reliable and valid diagnostic instrument (Adam et al., 2019).
OCD-related problem list (OCD-PL; Goletz, Adam & Döpfner, 2020). The OCD-related problem list exists in a parent form (children ≥ 4 years) and self-report form (adolescents ≥ 11 years). At pre-treatment (t0), the therapist completed the OCD-PL together with the patient and parents. Individual obsessions, unpleasant feelings (e.g. anxiety) and compulsions were written down. These individual OCD symptoms were then rated by patient and parents regarding frequency ("0 = not at all" to "4 = very much"), strain ("0 = no problem

at all" to "9 = it could not have been worse") and psychosocial impairment in school/job, leisure time and family life ("0 = not all" to "4 = extremely impaired") referring to the last week. To evaluate treatment effects, means of the weekly ratings regarding frequency, strain, and psychosocial impairment were used.

Daily Observation (Goletz, Döpfner & Roessner, 2018). This protocol includes columns regarding (1) time, (2) triggering events/obsessions, (3) extent of negative emotions (e.g. anxiety) on a scale from 0 to 100, (4) compulsions, (5) duration in minutes, (6) strain caused by OCD symptoms on a scale from 0 to 100. The OCD symptoms were recorded by patients (≥ 11 years) and parents separately on one weekday (*Daily Observation weekday*) and one day at the weekend (*Daily Observation weekend*) at pre-treatment (t0 and t1) and every treatment week. For the analyses, means of the extent of negative emotions, sum of duration, and means of strain of each rating were used.

OCD-functional impairment list (OCD-FL). The OCD-FL is based on the Weiss Functional Impairment Rating Scale – Parent Report (WFIRS-P, Canadian Attention Deficit Hyperactivity Disorder Resource Alliance (CADDRA), 2011). The OCD-FL includes 26 items and exists in a parent form (patient ≥ 6 years) and a self-report form (patients ≥ 11 years), which are constructed analogously to each other. Psychosocial impairment is assessed on a 4-point scale ranging from "0 = not at all" to "3 = very often or very much" with regard to five domains: (1) family, (2) learning & school, (3) life skills, (4) self-concept, (5) social activities. The total score was used for the analyses. As psychometric properties of the OCD-FL have not been evaluated so far, Cronbach's alpha for the total scale was computed using the study sample. Internal consistencies were good to excellent (self-report form: α = .90, parent form: α = .84).

Youth Self Report – YSR/ 11-18R (YSR; Döpfner et al., 2014) & Child Behavior Checklist/ 6-18R (CBCL; Döpfner et al., 2014). These instruments were originally developed by Achenbach & Rescorla (2001). The self-report (YSR: 112 items; patients ≥ 11 years) and parent report (CBCL: 113 items; patients ≥ 6 years) assess a range of behavioral and emotional problems in children and adolescents. Each item is rated on a 3-point scale ("0 = not true", "1 = somewhat or sometimes true", "2 = very true or often true"). Items are assigned to two broad-band syndrome scales (externalizing and internalizing problems) and eight syndrome scales (aggressive behavior, anxious/depressed, attention problems, rule-breaking behavior, somatic complaints, social problems, thought problems, withdrawn/depressed) and a total scale. Research has demonstrated good reliability and factorial validity (Döpfner et al., 2014). To evaluate overall comorbid symptoms, the broad-band syndrome scales and the total scale were used.

German Symptom Checklists for Anxiety Disorders and Obsessive-Compulsive Disorders (FBB-/SBB-ANZ; Döpfner et al., 2008). These questionnaires include the same 33 items

each, with 31 items assessing anxiety symptoms and two items assessing obsession and compulsion. All items are rated on a 4-point scale (“0 = not at all” to “3 =very much”). Furthermore, the questionnaires each include eight items assessing competences regarding sociability and confidence (scale: competences). Results from psychometric evaluations of the SBB-/FBB-ANZ supported reliability and validity (Döpfner et al. 2008). For the analyses, the total anxiety scale and the competence scale were used.

German Symptom Checklists for Depressive Disorders (FBB-/SBB-DES; Döpfner et al., 2008). The structure, implementation, and assessment are the same as described for the SBB-/FBB-ANZ. The total score scale includes 29 items, and a further eight items asked about competences regarding self-confidence and the ability to enjoy things (scale: competences). Research has yielded good results regarding reliability and validity (Döpfner et al. 2008). For the analyses, the total anxiety scale and the competence scale were used.

References

Abteilung für Neuropsychologie des Universitätsklinikums Bonn (2002). *Padua Inventory – Washington State University Revised (PI-WSUR).* Deutsche Übersetzung. Universität Bonn.

Achenbach, T. M. & Rescorla, L. A. (2001). *Manual for the ASEBA school-age forms & profiles: an integrated system of multi-informant assessment.* Burlington: University of Vermont, Research Center for Children, Youth & Famlies.

Adam, J., Goletz, H., Mattausch, S.-K., Plück, J. & Döpfner, M. (2019). Psychometric evaluation of a parent-rating and self-rating inventory for pediatric obsessive-compulsive disorder: German OCD Inventory for Children and Adolescents (OCD-CA). *Child and Adolescent Psychiatry and Mental Health, 13*, 1-13, doi: 10.1186/s13034-019-0286-z.

Burns, G. L., Keortge, S. G., Formea, G. M. & Sternberger, L. G. (1996). Revision of the Padua Inventory of obsessive compulsive disorder symptoms: Distinction between worry, obsessions, and compulsions. *Behaviour Research and Therapy, 34*, 163-173.

Canadian Attention Deficit Hyperactivity Disorder Resource Alliance (CADDRA). (2011). *Canadian ADHD Practice Guidelines (CAP-Guidelines)*. Retrieved 6 July 2020, from http://caddra.ca/pdfs/caddraGuidelines2011.pdf.

Döpfner, M., Görtz-Dorten, A., Lehmkuhl, G., Breuer, D. & Goletz, H. (2008). *Diagnostik-System für psychische Störungen nach ICD-10 und DSM-IV für Kinder und Jugendliche-II (DISYPS-II)*. Bern: Huber.

Döpfner M, Plueck J, Kinnen C & Arbeitsgruppe Deutsche Child Behavior Checklist. (2014). *CBCL Handbuch-Schulalter. Manual zum Elternfragebogen über das Verhalten von Kindern und Jugendlichen, (CBCL/ 6-18R), zum Lehrerfragebogen über das Verhalten von Kindern und Jugendlichen (TRF/6-18R) und zum Fragebogen für Jugendliche (YSR/11-18R)*. Goettingen: Hogrefe.

Goodman, W. K., Rasmussen, S. A., Price, L. H., Mazure, C., Rapoport, J. L., Heninger, G. R. & Charney, D. S. (1986). *Children's Yale-Brown Obsessive Compulsive Scale (CY-BOCS)*. Unpublished scale.

Goletz, H., Adam, J. & Döpfner, M. (2020). *DZ-KJ. Diagnostikum für Zwangsstörungen im Kindes- und Jugendalter*. Göttingen: Hogrefe.

Goletz, H. & Döpfner, M. (2018). Die klinische Beurteilung von Zwangssymptomen bei Kindern und Jugendlichen. Eine Studie mit der Children's Yale-Brown Obsessive-Compulsive Scale (CY-BOCS-D). *Zeitschrift für Kinder- und Jugendpsychiatrie und Psychotherapie*, 1-15, doi: 10.1024/1422-4917/a000642.

Goletz, H., Döpfner, M. & Roessner, V. (2018). Zwangsstörungen. *Leitfaden Kinder- und Jugendpsychotherapie*. Göttingen: Hogrefe.
