# Supplementary material for: Extended treatment of multimodal cognitive behavioral therapy in children and adolescents with obsessive–compulsive disorder improves symptom reduction: a within-subject design
Source: Child Adolesc Psychiatry Ment Health. 2022 Dec 9;16:99. doi: 10.1186/s13034-022-00537-z (PMC9737735; doi:10.1186/s13034-022-00537-z)
Supplement: Supplementary file 4 — Additional file 4. Individual end of treatment. The individual end of treatment as well as dropouts are presented in a figure. [file 13034_2022_537_MOESM4_ESM.pdf]

Additional file 4

Individual end of treatment

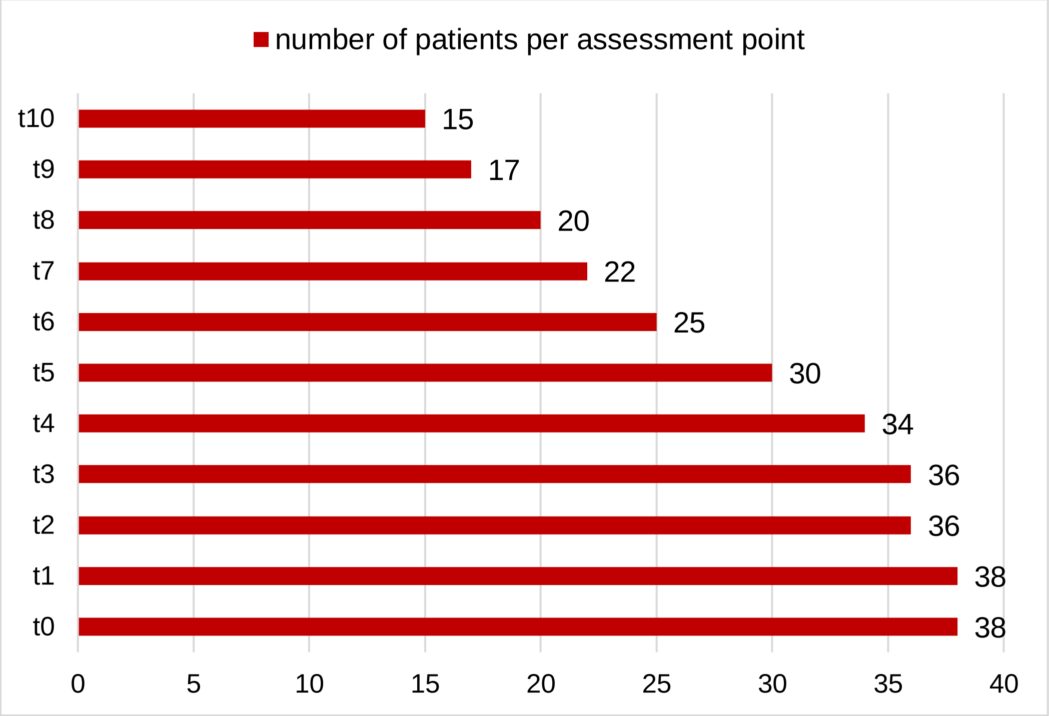

Note: *two dropouts at t2, two dropouts at t4, one dropout at t8*
