# Supplementary material for: Extended treatment of multimodal cognitive behavioral therapy in children and adolescents with obsessive–compulsive disorder improves symptom reduction: a within-subject design
Source: Child Adolesc Psychiatry Ment Health. 2022 Dec 9;16:99. doi: 10.1186/s13034-022-00537-z (PMC9737735; doi:10.1186/s13034-022-00537-z)
Supplement: Supplementary file 5 — Additional file 5. Results of multilevel analyses: Assessment (t0-t1) vs. treatment (t1-t3) vs. extended treatment (t3-t10). Changes during assessment phase and the treatment phases regarding the daily observation are shown in a table. [file 13034_2022_537_MOESM5_ESM.pdf]

Additional file 5

Results of multilevel analyses: Assessment (t0-t1) vs. treatment (t1-t3) vs. extended treatment (t3-t10)

		Change during assessment		Change during standard treatment		Change during extended treatment							
		phase 1: t0-t1		phase 2 and 3: t1-t3		phase 3a: t3-t5		phase 3b: t5-t7		phase 3c: t7-t9		phase 3d : t9-t10	
Outcome	*n*	*β (CI 95%)*	*ES*	*β (CI 95%)*	*ES*	*β (CI 95%)*	*ES*	*β (CI 95%)*	*ES*	*β (CI 95%)*	*ES*	*β (CI 95%)*	*ES*
Daily observation, weekday													
Extent of negative emotions	[29]	[-0.38^{a}]<br>[(-2.06 to 1.30)]	[-0.09]	[-1.68***^{b,c}]<br>[(-2.19 to -1.17)]	[-0.83]	[-0.50*^{a,d}]<br>[(-0.94 to -0.06)]	[-0.25]	[-0.44^{a,d}]<br>[(-0.92 to 0.05)]	[-0.22]	[-0.24^{a,d}]<br>[(-0.85 to 0.36)]	[-0.12]	[1.11^{a,d}]<br>[(-0.70 to 2.91)]	[0.27]
	{29}	{-0.67^{a}}<br>{(-2.06 to 0.72)}	{-0.19}	{-0.78**^{a,c}}<br>{(-1.33 to -0.23)}	{-0.45}	{-0.84***^{a,c}}<br>{(-1.35 to -0.33)}	{-0.49}	{0.69*^{b,d}}<br>{(0.13 to 1.25)}	{0.40}	{-0.56^{b,c}}<br>{(-1.26 to 0.15)}	{-0.33}	{0.19^{a,c}}<br>{(-1.93 to 2.31)}	{0.05}
Duration	[30]	[2.51^{a}]<br>[(-2.20 to 7.22)]	[0.15]	[-3.71***^{b,c}]<br>[(-5.19 to -2.23)]	[-0.44]	[-2.28***^{b,d}]<br>[(-3.56 to -1.01)]	[-0.27]	[0.01^{b,d}]<br>[(-1.39 to 1.42)]	[0.00]	[-0.78^{b,d}]<br>[(-2.54 to 0.98)]	[-0.09]	[0.29^{a,c}]<br>[(-5.02 to 5.59)]	[0.02]
	{33}	{-0.10^{a}}<br>{(-3.66 to 3.45)}	{-0.01}	{-3.72***^{b,c}}<br>{(-5.11 to -2.32)}	{-0.52}	{-0.53^{a,d}}<br>{(-1.82 to 0.76)}	{-0.07}	{1.31^{a,d}}<br>{(-0.15 to 2.76)}	{0.18}	{-1.91*^{b,d}}<br>{(-3.72 to -0.11)}	{-0.27}	{0.88^{a,c}}<br>{(-4.40 to 6.16)}	{0.06}
Strain	[29]	[0.91^{a}]<br>[(-0.69 to 2.50)]	[0.21]	[-1.54***^{b,c}]<br>[(-2.04 to -1.04)]	[-0.73]	[-0.91***^{b,d}]<br>[(-1.35 to -0.48)]	[-0.43]	[-0.69**^{b,d}]<br>[(-1.17 to -0.21)]	[-0.33]	[-0.37^{b,d}]<br>[(-0.98 to 0.23)]	[-0.18]	[1.19^{a,d}]<br>[(-0.63 to 3.00)]	[0.28]
	{32}	{-2.71***^{a}}<br>{(-4.27 to -1.15)}	{-0.77}	{-0.93**^{b,c}}<br>{(-1.54 to -0.33)}	{-0.53}	{-0.76**^{b,c}}<br>{(-1.31 to -0.22)}	{-0.43}	{0.79**^{b,d}}<br>{(0.16 to 1.42)}	{0.45}	{-1.22**^{b,c}}<br>{(-2.05 to -0.39)}	{-0.69}	{-0.34^{a,c}}<br>{(-2.77 to 2.09)}	{-0.10}
Daily observation, weekend													
Extent of negative emotions	[28]	[-1.12^{a}]<br>[(-2.98 to 0.75)]	[-0.30]	[-1.20***^{a,c}]<br>[(-1.71 to -0.68)]	[-0.65]	[-0.73***^{a,d}]<br>[(-1.17 to -0.28)]	[-0.39]	[-0.61**^{b,d}]<br>[(-1.10 to -0.12)]	[-0.33]	[-0.30^{b,d}]<br>[(-0.92 to 0.32)]	[-0.16]	[0.37^{a,c}]<br>[(-1.53 to 2.27)]	[0.10]
	{29}	{-1.68*^{a}}<br>{(-3.23 to -0.13)}	{-0.43}	{-0.75*^{b,c}}<br>{(-1.37 to -0.14)}	{-0.39}	{-0.15^{b,d}}<br>{(-0.70 to 0.41)}	{-0.08}	{0.03^{b,d}}<br>{(-0.55 to 0.62)}	{0.02}	{0.04^{b,d}}<br>{(-0.70 to 0.78)}	{0.02}	{-1.40^{a,c}}<br>{(-3.65 to 0.84)}	{-0.36}
Duration	[30]	[0.58^{a}]<br>[(-5.15 to 6.32)]	[0.04]	[-2.20**^{b,c}]<br>[(-3.74 to -0.65)]	[-0.33]	[-2.98***^{b,c}]<br>[(-4.32 to -1.64)]	[-0.45]	[0.47^{a,d}]<br>[(-1.01 to 1.96)]	[0.07]	[-1.00^{a,c}]<br>[(-2.88 to 0.89)]	[-0.15]	[-0.31^{a,c}]<br>[(-6.14 to 5.52)]	[-0.02]
	{31}	{-3.62^{a}}<br>{(-7.72 to 0.48)}	{-0.27}	{-3.86***^{a,c}}<br>{(-5.36 to -2.36)}	{-0.57}	{-0.50^{b,d}}<br>{(-1.87 to 0.87)}	{-0.07}	{1.17^{b,d}}<br>{(-0.36 to 2.70)}	{0.17}	{-1.34^{b,d}}<br>{(-3.35 to 0.66)}	{-0.20}	{0.25^{a,c}}<br>{-5.66 to 6.16}	{0.02}
Strain	[29]	[0.75^{a}]<br>[(-1.25 to 2.77)]	[0.17]	[-1.48***^{b,c}]<br>[(-1.20 to -0.97)]	[-0.65]	[-1.02***^{b,d}]<br>[(-1.47 to -0.58)]	[-0.45]	[-0.68**^{b,d}]<br>[(-1.18 to -0.18)]	[-0.30]	[-0.55^{b,d}]<br>[(-1.18 to 0.08)]	[-0.24]	[0.69^{a,d}]<br>[(-1.29 to 2.67)]	[0.15]
	{30}	{-2.94***^{a}}<br>{(-4.72 to -1.17)}	{-0.97}	{-1.14***^{b,c}}<br>{(-1.80 to -0.49)}	{-0.75}	{-0.32^{b,d}}<br>{(-0.91 to 0.27)}	{-0.21}	{0.42^{b,d}}<br>{(-0.24 to 1.09)}	{0.28}	{-0.87*^{b,c}}<br>{(-1.76 to 0.01)}	{-0.57}	{-1.42^{a,c}}<br>{(-4.03 to 1.20)}	{-0.47}

Note: *n* = sample size, *β* = slope, *CI* = confidence interval, *ES* = effect size; clinical rating, [self-report], {parent report}; *p ≤ .05, **p ≤ .01, ***p ≤ .001; ^{a,b,c,d} slopes with superscripts (a) do not differ significantly from assessment phase, slopes with superscript (b) differ significantly at a level of ≤ .05 from assessment phase; slopes with superscripts (c) do not differ significantly from standard treatment phase, slopes with superscript (d) differ significantly at a level of ≤ .05 from standard treatment phase
