# Supplementary material for: Extended treatment of multimodal cognitive behavioral therapy in children and adolescents with obsessive–compulsive disorder improves symptom reduction: a within-subject design
Source: Child Adolesc Psychiatry Ment Health. 2022 Dec 9;16:99. doi: 10.1186/s13034-022-00537-z (PMC9737735; doi:10.1186/s13034-022-00537-z)
Supplement: Supplementary file 6 — Additional file 6. Results of multilevel analyses: Assessment (t0-t1) vs. non-exposure CBT (t1-t2) vs. exposure CBT (t2-t3) vs. extended treatment (t3-t10). Changes during assessment phase and the treatment phases as well as effects regarding the clinician-rated OCD severity, patient- and parent-rated OCD symptoms and OCD-related problems are presented in a table. [file 13034_2022_537_MOESM6_ESM.pdf]

## Additional file 6

*Results of multilevel analyses: Assessment (t0-t1) vs. non-exposure CBT (t1-t2) vs. exposure CBT (t2-t3) vs. extended treatment (t3-t10)*

| | | Change during assessment (phase 1: t0-t1) | | | | Change during non-exposure CBT (phase 2a: t1-t2) | | | | Change during exposure CBT (phase 2b: t2-t3) | | | | Exposure CBT effect |
|---|---|---|---|---|---|---|---|---|---|---|---|---|---|---|
| Outcome | *n* | *β* | *CI (95%)* | ≤ *p* | *ES* | *β* | *CI (95%)* | ≤ *p* | *ES* | *β* | *CI (95%)* | ≤ *p* | *ES* | *Δ* $ES_{NE\text{-}E}$ |
| **CY-BOCS-D rating scale** | | | | | | | | | | | | | | |
| **Total OCD severity** | **38** | **-0.37$^{a}$** | **-0.74 to -0.00** | **.050** | **-0.52** | **-0.46$^{a,c}$** | **-0.84 to -0.09** | **.016** | **-0.65** | **-0.62$^{a,c}$** | **-0.98 to -0.26** | **.001** | **-0.87** | **0.22** |
| Obsession severity | 38 | -0.18$^{a}$ | -0.41 to 0.05 | .119 | -0.25 | -0.24$^{a,c}$ | -0.47 to -0.00 | .047 | -0.33 | -0.24$^{a,c}$ | -0.46 to -0.01 | .041 | -0.33 | -0.00 |
| Compulsion severity | 38 | -0.21$^{a}$ | -0.40 to -0.03 | .025 | -0.57 | -0.19$^{a,c}$ | -0.38 to -0.00 | .046 | -0.52 | -0.38$^{a,d}$ | -0.56 to -0.20 | .001 | -1.03 | 0.50 |
| OCD-CA | | | | | | | | | | | | | | |
| Total OCD symptoms | [31] | [-0.38$^{a}$] | [-1.30 to 0.54] | [.414] | [-0.14] | [-0.34$^{a,c}$] | [-1.25 to 0.57] | [.466] | [-0.12] | [-0.72$^{a,c}$] | [-1.60 to -0.16] | [.108] | [-0.26] | [0.14] |
| | {37} | {-0.88$^{a}$} | {-1.67 to -0.09} | {.030} | {-0.31} | {-0.09$^{b,c}$} | {-0.89 to 0.71} | {.825} | {-0.03} | {-1.03$^{a,d}$} | {-1.82 to -0.24} | {.011} | {-0.36} | {0.33} |
| OCD-related problem list | | | | | | | | | | | | | | |
| Frequency | [31] | [-0.05$^{a}$] | [-0.09 to -0.01] | [.025] | [-0.28] | [-0.03$^{a,c}$] | [-0.07 to 0.00] | [.056] | [-0.19] | [-0.06$^{a,d}$] | [-0.08 to -0.03] | [.001] | [-0.35] | [0.16] |
| | {32} | {-0.00$^{a}$} | {-0.05 to 0.04} | {.880} | {-0.03} | {-0.06$^{b,c}$} | {-0.10 to -0.02} | {.002} | {-0.49} | {-0.06$^{b,c}$} | {-0.09 to -0.02} | {.002} | {-0.42} | {-0.07} |
| Strain | [31] | [-0.14$^{a}$] | [-0.24 to -0.05] | [.003] | [-0.47] | [-0.02$^{b,c}$] | [-0.10 to 0.06] | [.665] | [-0.06] | [-0.11$^{a,d}$] | [-0.17 to -0.05] | [.001] | [-0.35] | [0.29] |
| | {32} | {-0.14$^{a}$} | {-0.27 to -0.02} | {.022} | {-0.55} | {-0.07$^{a,c}$} | {-0.18 to 0.03} | {.186} | {-0.27} | {-0.13$^{a,c}$} | {-0.22 to -0.04} | {.004} | {-0.51} | {0.24} |
| Psychosocial impairment | [30] | [-0.06$^{a}$] | [-0.10 to -0.02] | [.001] | [-0.36] | [-0.03$^{b,c}$] | [-0.06 to -0.00] | [.038] | [-0.18] | [-0.03$^{b,c}$] | [-0.05 to -0.01] | [.008] | [-0.18] | [-0.00] |
| | {36} | {-0.03$^{a}$} | {-0.07 to 0.01} | {.168} | {-0.24} | {-0.06$^{a,c}$} | {-0.10 to -0.02} | {.003} | {-0.47} | {-0.05$^{a,c}$} | {-0.08 to -0.01} | {.006} | {-0.36} | {-0.11} |

*Note*: *n* = sample size, *β* = slope, *CI* = confidence interval, *p* = significance value, *ES* = effect size, *Δ* $ES_{NE\text{-}E}$ = difference between the effect size of the non-exposure CBT phase (NE) and the effect size of the exposure CBT phase (E); clinical rating, [self-report], {parent report}; *p ≤ .05, **p ≤ .01, ***p ≤ .001; $^{a,b,c,d}$ slopes with superscripts (a) do not differ significantly from assessment phase, slopes with superscript (b) differ significantly at a level of ≤ .05 from assessment phase; slopes with superscripts (c) do not differ significantly from non-exposure CBT phase, slopes with superscript (d) differ significantly at a level of ≤ .05 from non-exposure CBT phase
