# Supplementary material for: Extended treatment of multimodal cognitive behavioral therapy in children and adolescents with obsessive–compulsive disorder improves symptom reduction: a within-subject design
Source: Child Adolesc Psychiatry Ment Health. 2022 Dec 9;16:99. doi: 10.1186/s13034-022-00537-z (PMC9737735; doi:10.1186/s13034-022-00537-z)
Supplement: Supplementary file 7 — Additional file 7. Results of multilevel analyses: Assessment (t0-t1) vs. non-exposure CBT (t1-t2) vs. exposure CBT (t2-t3) vs. extended treatment (t3-t10). Changes during assessment phase and the treatment phases as well as effects regarding daily observation are shown in a table. [file 13034_2022_537_MOESM7_ESM.pdf]

Additional file 7

Results of multilevel analyses: Assessment (t0-t1) vs. non-exposure CBT (t1-t2) vs. exposure CBT (t2-t3) vs. extended treatment (t3-t10)

		Change during assessment (phase 1: t0-t1)				Change during non-exposure CBT (phase 2a: t1-t2)				Change during exposure CBT (phase 2b: t2-t3)				Exposure CBT effect
Outcome	*n*	*β*	*CI (95%)*	*≤ p*	*ES*	*β*	*CI (95%)*	*≤ p*	*ES*	*β*	*CI (95%)*	*≤ p*	*ES*	ΔES_{NE-E}
Daily observation, weekday														
Extent of negative emotions	[29]	[-0.70[a]]	[-2.49 to 1.10]	[.446]	[-0.17]	[-1.04[a,c]]	[-2.41 to 0.33]	[.138]	[-0.26]	[-2.14[b,d]]	[-3.18 to -1.10]	[.001]	[-0.53]	[0.27]
	{29}	{-0.94[a]}	{-2.42 to 0.54}	{.212}	{-0.27}	{-0.14[a,c]}	{-1.47 to 1.19}	{.836}	{-0.04}	{-1.31[a,d]}	{-2.44 to -0.17}	{.024}	{-0.38}	{0.34}
Duration	[30]	[4.18[a]]	[-0.84 to 9.20]	[.103]	[0.25]	[-7.19[b,c]]	[-11.13 to -3.24]	[.001]	[-0.42]	[-1.20[b,d]]	[-4.22 to 1.83]	[.438]	[-0.07]	[-0.35]
	{33}	{-0.34[a]}	{-4.13 to 3.44}	{.859}	{-0.02}	{-3.14[a,c]}	{-6.52 to 0.24}	{.069}	{-0.22}	{-4.18[b,c]}	{-7.03 to -1.34}	{.004}	{-0.29}	{0.07}
Strain	[29]	[0.97[a]]	[-0.73 to 2.66]	[.264]	[0.23]	[-1.66[b,c]]	[-2.99 to -0.34]	[.014]	[-0.39]	[-1.45[b,c]]	[-2.48 to -0.43]	[.006]	[-0.34]	[-0.05]
	{32}	{-2.98[a]}	{-4.66 to -1.29}	{.001}	{-0.84}	{-0.34[b,c]}	{-1.84 to 1.16}	{.654}	{-0.10}	{-1.38[b,c]}	{-2.58 to -0.18}	{.024}	{-0.39}	{0.29}
Daily observation, weekend														
Extent of negative emotions	[28]	[-1.46[a]]	[-3.43 to 0.51]	[.145]	[-0.40]	[-0.50[a,c]]	[-1.86 to 0.87]	[.474]	[-0.13]	[-1.70[a,d]]	[-2.74 to -0.66]	[.001]	[-0.46]	[0.32]
	{29}	{-2.00[a]}	{-3.67 to -0.33}	{.019}	{-0.52}	{-0.04[b,c]}	{-1.53 to 1.45}	{.955}	{-0.01}	{-1.32[a,d]}	{-2.56 to -0.07}	{.038}	{-0.34}	{0.33}
Duration	[30]	[0.87[a]]	[-5.14 to 6.88]	[.776]	[0.07]	[-2.81[a,c]]	[-6.91 to 1.30]	[.180]	[-0.21]	[-1.75[a,c]]	[-4.91 to 1.40]	[.276]	[-0.13]	[-0.08]
	{31}	{-3.68[a]}	{-8.00 to 0.64}	{.095}	{-0.27}	{-3.71[a,c]}	{-7.33 to -0.09}	{.045}	{-0.27}	{-3.99[a,c]}	{-7.14 to -0.84}	{.013}	{-0.29}	{0.02}
Strain	[29]	[0.65[a]]	[-1.45 to 2.74]	[.545]	[0.14]	[-1.24[b,c]]	[-2.59 to 0.11]	[.073]	[-0.27]	[-1.66[b,c]]	[-2.71 to -0.61]	[.002]	[-0.36]	[0.09]
	{30}	{-2.83[a]}	{-4.72 to -0.93}	{.004}	{-0.93}	{-1.41[a,c]}	{-3.07 to 0.25}	{.095}	{-0.46}	{-0.93[b,c]}	{-2.31 to 0.45}	{.186}	{-0.31}	{-0.16}

Note: *n* = sample size, *β* = slope, *CI* = confidence interval, *p* = significance value, *ES* = effect size, ΔES_{NE-E} = difference between the effect size of the non-exposure CBT phase (NE) and the effect size of the exposure CBT phase (E); clinical rating, [self-report], {parent report}; *p ≤ .05, **p ≤ .01, ***p ≤ .001; [a,b,c,d] slopes with superscripts (a) do not differ significantly from assessment phase, slopes with superscript (b) differ significantly at a level of ? .05 from assessment phase; slopes with superscripts (c) do not differ significantly from non-exposure CBT phase, slopes with superscript (d) differ significantly at a level of ≤ .05 from non-exposure CBT phase
