# Supplementary material for: Extended treatment of multimodal cognitive behavioral therapy in children and adolescents with obsessive–compulsive disorder improves symptom reduction: a within-subject design
Source: Child Adolesc Psychiatry Ment Health. 2022 Dec 9;16:99. doi: 10.1186/s13034-022-00537-z (PMC9737735; doi:10.1186/s13034-022-00537-z)
Supplement: Supplementary file 8 — Additional file 8. Results of multilevel analyses: Assessment (t0-t1) vs. non-exposure CBT (t1-t2) vs. exposure CBT (t2-t3) vs. extended treatment (t3-t10). Changes during assessment phase and the treatment phases as well as effects regarding OCD functional impairment are presented in a table. [file 13034_2022_537_MOESM8_ESM.pdf]

Additional file 8

Results of multilevel analyses: Assessment (t0-t1) vs. non-exposure CBT (t1-t2) vs. exposure CBT (t2-t3) vs. extended treatment (t3-t10)

		Change during assessment (phase 1: t0-t1)				Change during non-exposure CBT (phase 2a: t1-t2)				Change during exposure CBT (phase 2b: t2-t3)				Exposure CBT effect
Outcome	*n*	*β*	*CI (95%)*	≤ *p*	*ES*	*β*	*CI (95%)*	≤ *p*	*ES*	*β*	*CI (95%)*	≤ *p*	*ES*	ΔES_{NE-E}
OCD functional impairment list														
Total impairment	[30]	[-1.33[a]]	[-1.65 to -1.01]	[.001]	[-0.57]	[-0.39[b,c]]	[-0.65 to -0.14]	[.002]	[-0.17]	[-0.34[b,c]]	[-0.54 to -0.14]	[.001]	[-0.14]	[-0.02]
	{35}	{-1.51[a]}	{-2.03 to -0.98}	{.001}	{-0.79}	{-0.59[b,c]}	{-1.06 to -0.13}	{.013}	{-0.31}	{-0.54[b,c]}	{-0.94 to -0.13}	{.010}	{-0.28}	{-0.03}

Note: *n* = sample size, *β* = slope, *CI* = confidence interval, *p* = significance value, *ES* = effect size, ΔES_{NE-E} = difference between the effect size of the non-exposure CBT phase (NE) and the effect size of the exposure CBT phase (E); clinical rating, [self-report], {parent report}; *p ≤ .05, **p ≤ .01, ***p ≤ .001; [a,b,c,d] slopes with superscripts (a) do not differ significantly from assessment phase, slopes with superscript (b) differ significantly at a level of ≤ .05 from assessment phase; slopes with superscripts (c) do not differ significantly from non-exposure CBT phase, slopes with superscript (d) differ significantly at a level of ≤ .05 from non-exposure CBT phase
