# Supplementary material for: Extended treatment of multimodal cognitive behavioral therapy in children and adolescents with obsessive–compulsive disorder improves symptom reduction: a within-subject design
Source: Child Adolesc Psychiatry Ment Health. 2022 Dec 9;16:99. doi: 10.1186/s13034-022-00537-z (PMC9737735; doi:10.1186/s13034-022-00537-z)
Supplement: Supplementary file 9 — Additional file 9. Benchmarking: Study characteristics. Characteristics of efficacy studies (Meta-analyses) and effectiveness studies are summarized in a table for benchmarking. [file 13034_2022_537_MOESM9_ESM.pdf]

Additional file 9

Benchmarking: Study characteristics

	Efficacy studies (Meta-analyses)		Effectiveness studies				
Study	Sánchez-Meca et al. (2014)	McGuire et al. (2015)	Valderhaug et al. (2007)	Nakatani et al. (2009)	Farrell, Schlup & Boschen (2010)	Torp et al. (2015)	Current Study
Setting	varying, not described within meta-analysis	varying, not described within meta-analysis	outpatient community child and adolescent psychiatry	OCD clinic	outpatient private community-based specialist clinic	community mental health clinics	university-based outpatient clinic
Therapists	varying, not described within meta-analysis	varying, not described within meta-analysis	experienced psychologist and physicians	experienced cognitive behavioral therapists or trainees	postgraduate level therapists with some previous experience in treatment of OCD (at least one client treated in the past under supervision)	psychiatrists, clinical psychologists or certified psychotherapists with at least five years of clinical experience	trainees as child and adolescent psychotherapists
Training / supervision	varying, not described within meta-analysis	varying, not described within meta-analysis	intensive 4-day training workshop and 2-5 h case discussions and supervision every third week	no training reported, trainees receive detailed supervision	training consisted of observing a group treatment program conducted by the first author, about 2h of informal weekly group supervision	therapists with little experience in OCD treatment received ten days of training, monthly 3h group supervision	no special training, weekly 2h group supervision
n of studies	11 CBT studies	10 CBT studies					
Sample size *n*	472 participants	507 participants	28 participants	75 participants	35 participants	269 participants	38 participants
Age in years: *range, M (SD)*	*M* = 12.1	*Range of means*: 5.8 - 14.6	8-17, 13.3 (2.3)	8-18, 13.7 (2.4)	7-17, 12.3 (2.6)	7-17, 12.8 (2.7)	6-20, 13.3 (3.6)
Gender: *male %*	50.6	not reported	50	56	57.6	48.7	39.5
Pre CY-BOCS total score *mean (SD)*	not reported	*Range of means*: 22.1 - 25.6	23.1 (3.1)	22.6 (5.2)	23.5 (5.8)	24.6 (5.1)	25.05 (4.26)
Comorbidity rates *(%)*	Range: 40.8 - 96.7 (data for one study is not available)	not reported as overall comorbidity rates	62.5	36	54	40.5	23.68
OCD-specific medication *(%)*	not reported	*Range of means*: 0.0 - 55.0 (only SRI medication is reported)	8.3	77.3	17.1	not reported	10.5
Treatment duration	*M* = 11.8 weeks	Range: 9-14 sessions	12 sessions (10 weekly sessions, last 2 sessions were conducted every second week)	*M* = 11 sessions (*SD* = 5, range 5-28)	*M* = 11.5 (*SD* = 1.3), range: 8-14 sessions individually or in small groups	14 weekly sessions	*M* = 41.1 (*SD* = 14.2), range: 18-54 weekly sessions
Treatment intensity	*M* = 1.2 h per week (data for one study is not available)	1h per session	about 45 min per session	about 1h per session	approximately 1h or 1.5h for groups	75 min per session	50-100 min per session
